# epiArt: a graphical HLA eplet amino acid repertoire translation reveals the need for an epitope driven revision of allele group nomenclature

**DOI:** 10.3389/fgene.2024.1449301

**Published:** 2024-10-16

**Authors:** Ilias Doxiadis, Claudia Lehmann, Nils Lachmann, Henry Loeffler-Wirth

**Affiliations:** ^1^ Laboratory for Transplantation Immunology, Institute for Transfusion Medicine, University Hospital Leipzig, Leipzig, Germany; ^2^ Institute for Transfusion Medicine, H and I Laboratory, Charité-Universitätsmedizin Berlin, Berlin, Germany; ^3^ Interdisciplinary Centre for Bioinformatics, IZBI, Leipzig University, Leipzig, Germany

**Keywords:** HLA alleles, eplets, epitopes, HLA mismatches, epiart, confirmed epitopes, aminoacid polymorphism

## Abstract

**Introduction:**

The immune response after transplantation depends on recipient/donor HLA allele mismatches. To enhance our understanding of the relations of HLA alleles in terms of amino-acid polymorphisms and shared epitopes, we assessed pairwise sequence difference between HLA-alleles.

**Methods:**

We translated amino-acid sequences of confirmed eplets into an atlas of HLA class I and II antigens, followed by visualization of the pairwise allele distances by means of antigen-specific disparity graphs in differential amino-acid space. We obtained an overview of relationships of all alleles of an antigen, corresponding similarity/dissimilarity structures, outliers, alleles with similarity to different antigen groups. Additionally, we calculated prevalence of the amino-acids for each polymorphic sequence position and visualized them in amino-acid motif plots of all alleles belonging to an antigen.

**Results:**

Our visualizations show strongly varying intra-group heterogeneity of HLA class I and II alleles, as well as shared inter-group and inter-locus eplets and epitopes, indicating a benefit of epitope-based transplant matching: Single allele recipient/donor mismatches potentially refer to identical eplets, or to a set of multiple mismatched eplets.

**Discussion:**

This data reveals inconsistencies in the HLA group nomenclature and consequently adds a new level of quality to allocation, motivating the definition of tolerable or taboo mismatches.

## 1 Introduction

The products of the Major Histocompatibility Complex (MHC) are predominantly the targets for immune responses upon pregnancy, transfusion, and transplantation (Thorsby, 2009). These three routes are thought to be the major activation pathway. Cross-reactivity to viral or other infections has been described for the cellular component of the immune response (D’Orsogna et al., 2012). Scarcely data have been shown for cross-reactive humoral anti HLA antibodies against other structures (van Heemst et al., 2015). The immune response towards the non-self-allo-HLA-antigens depends, as already demonstrated, on the HLA make-up of the responder and the incompatible epitopes on the donor. Alignment of the HLA sequences defined to date are found on the IPD-IGMT/HLA database and the allele frequency net database, while the antibody-confirmed amino acid sequences are found on the HLA Eplet Registry (EpRegistry) (HLA, 2024b). We use the results of the German population presented in the allele frequencies database (AFND, 2020), and the data provided by the different groups worldwide attempted to visualize the “cross-reactivity” of the alleles of the HLA-A, HLA-B, HLA-C, HLA-DRB1, HLA-DRB345, HLA-DQA1, HLA-DQB1, HLA-DPA1 and HLA-DPB1 loci.

The term “eplet” denotes small configurations of few polymorphic amino acid on the HLA molecular surface (Duquesnoy, 2006). These eplets were collected in the HLA Eplet Registry (HLA, 2024b) and thoroughly characterized (see, e.g., (Bezstarosti et al., 2021; Meng et al., 2023)). Epitopes, on the other hand, can be defined as the specific antigenic determinant of an antigen that is recognized and bound by an antibody or a receptor on a T-cell. In general, epitopes are larger (15–22 amino acid molecules) than eplets (2-5 residues). We regard eplets as proxies determining the corresponding epitopes. Accordingly, we here use the term eplet in the context of the short amino acid sequences derived from the EpRegistry, and the term epitope for the antigenic determinant in general. The term “shared epitopes” was introduced to explain why some HLA antigens are recognized by the same antibody and is used for alleles with common eplets.

Within the HLA community, the term cross-reactivity has been used to term a serological reactivity between HLA antigens and a variety of antibodies. From our computational perspective, we use the term cross-reactivity when a particular HLA allele is most similar to one or more alleles of a different allele group by means of our metric and as visualized by the disparity graphs presented here.

In this report, we will analyze the polymorphic amino acid sequences of eplets and the pairwise sequence difference between HLA alleles. Therefore, we will translate amino acid sequences of all currently antibody-confirmed eplets into a sequence atlas comprising all classical HLA class I and II alleles. Their sequences will be transferred into a differential amino acid space, followed by visualization of the pairwise allele distances by means of antigen-specific disparity graphs. They will give an overview of relationships of all alleles of the antigen, corresponding similarity/dissimilarity structures, outlier alleles, and alleles with similarity to different antigen alleles.

## 2 Materials and methods

### 2.1 Eplet amino acid sequences and motif plots

Data on amino acid polymorphism and positions were obtained from the HLA Eplet Registry (HLA, 2024b) as of May 2020. We restrained our analyses to antibody-confirmed eplets, comprising 72 entries for class I, and 74 for class II. For each allele in the registry, we collected all associated eplets and marked their polymorphic residues at the corresponding positions. This way, we generated an atlas of polymorphic amino acids of all currently antibody-confirmed eplets as proxy for their antigenic epitopes.

For each polymorphic sequence position, we calculated prevalence of the different amino acids and visualized them in term of stacked bar plots, representing amino acid motif plots of all alleles belonging to an antigen, or of all alleles of a locus visualizing locus motifs.

### 2.2 Disparity graphs of HLA alleles based on polymorphic eplets

Our database contains polymorphic amino acid residues and positions for all eplets of the HLA class I and II alleles. Hamming distance of all pairs of each two alleles is computed by means of the number positions with of differing amino acid.

This numeric allele-by-allele differential amino acid residue matrix (adjacency matrix) can be translated into an allele similarity graph using R package “igraph” (Csardi and Nepusz, 2006). The resulting overall graph of all HLA alleles is then split into subgraphs for better visualization: These subgraphs contain only alleles of one main allele group as nodes, where each allele/node is connected to the most similar other allele/node via an edge (1-nearest-neighbour graph). In case of alleles, which are more similar to alleles of another group than to any allele of the own group, the corresponding inter-group cross-reactive alleles are also added to the subgraph as nodes and connected via edges.

## 3 Results

### 3.1 Eplet-derived polymorphic amino acid sequences of HLA class I alleles

**FIGURE 1:**
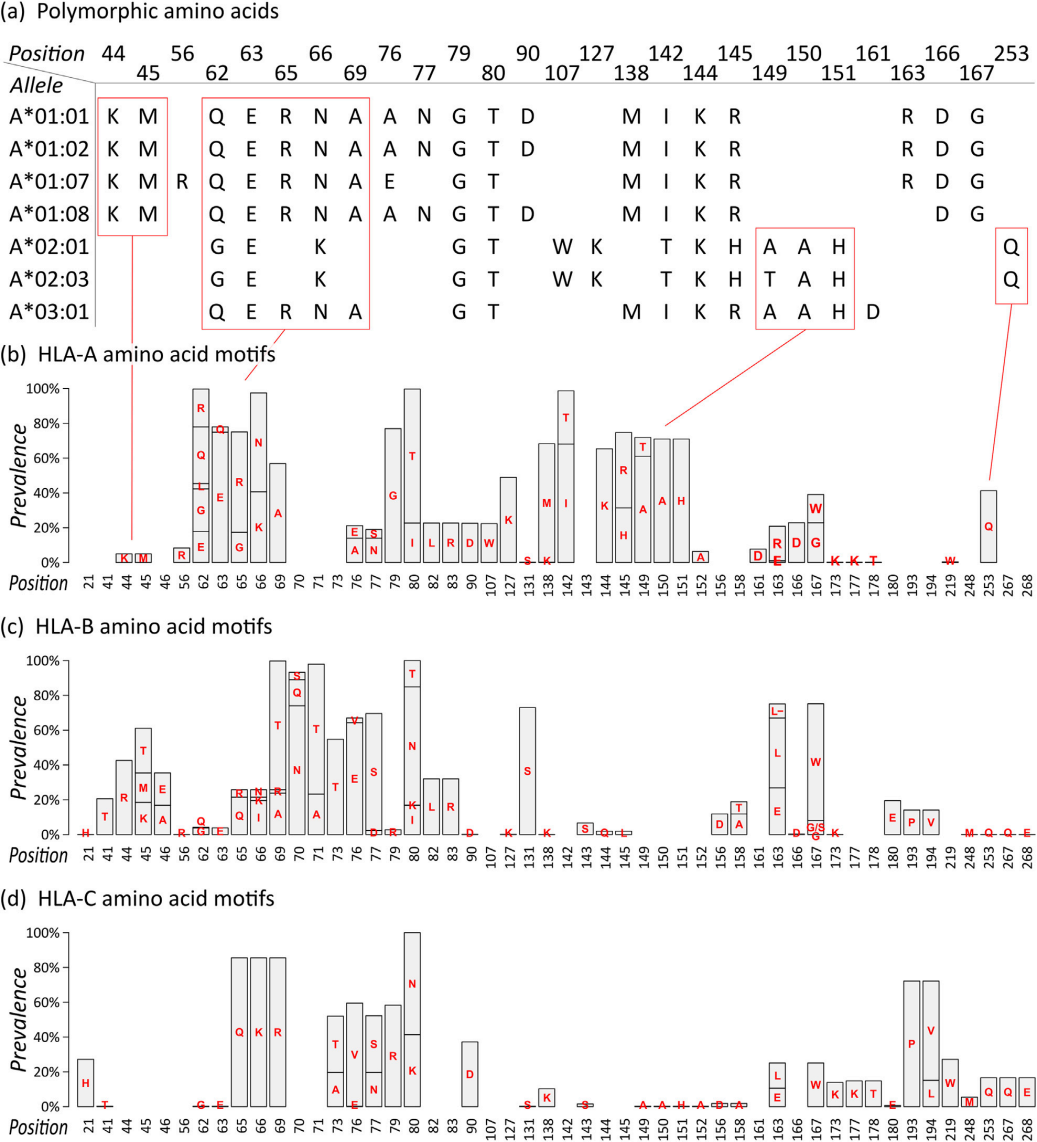
Eplet polymorphism of HLA class I alleles: **(A)** Polymorphic amino acid sequences of selected HLA-A alleles based on eplet information provided by EpRegistry database. For example, HLA-A*01:01 has antibody-confirmed eplets 44KM, 62QE, 65RNA, and further. Numbers specify the position in the mature protein, capital letters refer to the 1-letter amino acid symbols. Corresponding polymorphic amino acids are placed at the specified position, resulting in amino acid sequences for all alleles. **(B)** Motif plot of the polymorphic amino acid residues across the HLA-A alleles. Height of the bars represents prevalence of a particular amino acid in the group. Empty bars represent monomorphic amino acid positions. **(C–D)** Motif plots of HLA-B and -C, respectively.

For each allele, we extracted the polymorphic amino acid residues of their eplets, resulting in an amino acid sequence (Figure 1A; all sequences are given as Supplementary File S1). For example, HLA-A*01:01 features the following eplets: 44KM, 62QE, 65RNA, 76ANT, 79GT, 90D, 138MI, 144KR, 163RG, and 166DG. The numbers thereby specify the position of the amino acid in the mature protein, the letters refer to the 1-letter amino acid symbols. 44KM consequently refers to lysine and methionine at positions 44 and 45, respectively. The sequence of polymorphic amino acids of HLA-A*01:01 is generated by collecting all polymorphic residues from the 10 epitopes listed above.

The sequences of selected HLA-A alleles show characteristic patterns, for example, a common KM structure characteristic for all alleles in the A*01 group, a QUERNA structure found in most A*01, A*03, and A*11 alleles, and an AAH structure found in many HLA-A alleles except A*01 group (Figure 1A; Supplementary File S1). The data reflect the serologically found cross-reactivity within these allele groups (MacPherson, 1989). Also, monomorphic alleles can be identified as represented by their eplet sequences, for example, HLA-A*01:01 and HLA-A*01:02, which show identical (antibody-confirmed) eplets however differing by two not antibody-confirmed eplets 9S and 17S. In our study, we restrict to antibody-confirmed eplets reported in the EpRegistry as a proof of concept.

We then translated the sequences of all HLA-A alleles into a motif plot, where each bar reflects the prevalence of the different polymorphic amino acid residue in the mature HLA protein at a particular position (Figure 1B). It summarizes the patterns observed in the selected alleles described above, and all currently antibody-confirmed HLA-A alleles, forming the eplet amino acid repertoire of this locus.

Motif plots of HLA-B and -C were generated in the same manner (Figures 1C, D). The reveal mainly distinct and characteristic repertoires for these loci, showing that they generally form specific antibody binding sites. Only few and subtle commonalities can be observed, for example, the ILRD motif between positions 80 and 90, PV at 193 and 194 (HLA-B and -C only), and (M)QQE between 248 and 268 as previously reported (Vittoraki et al., 2021).

### 3.2 HLA-A allele disparity graphs

**FIGURE 2:**
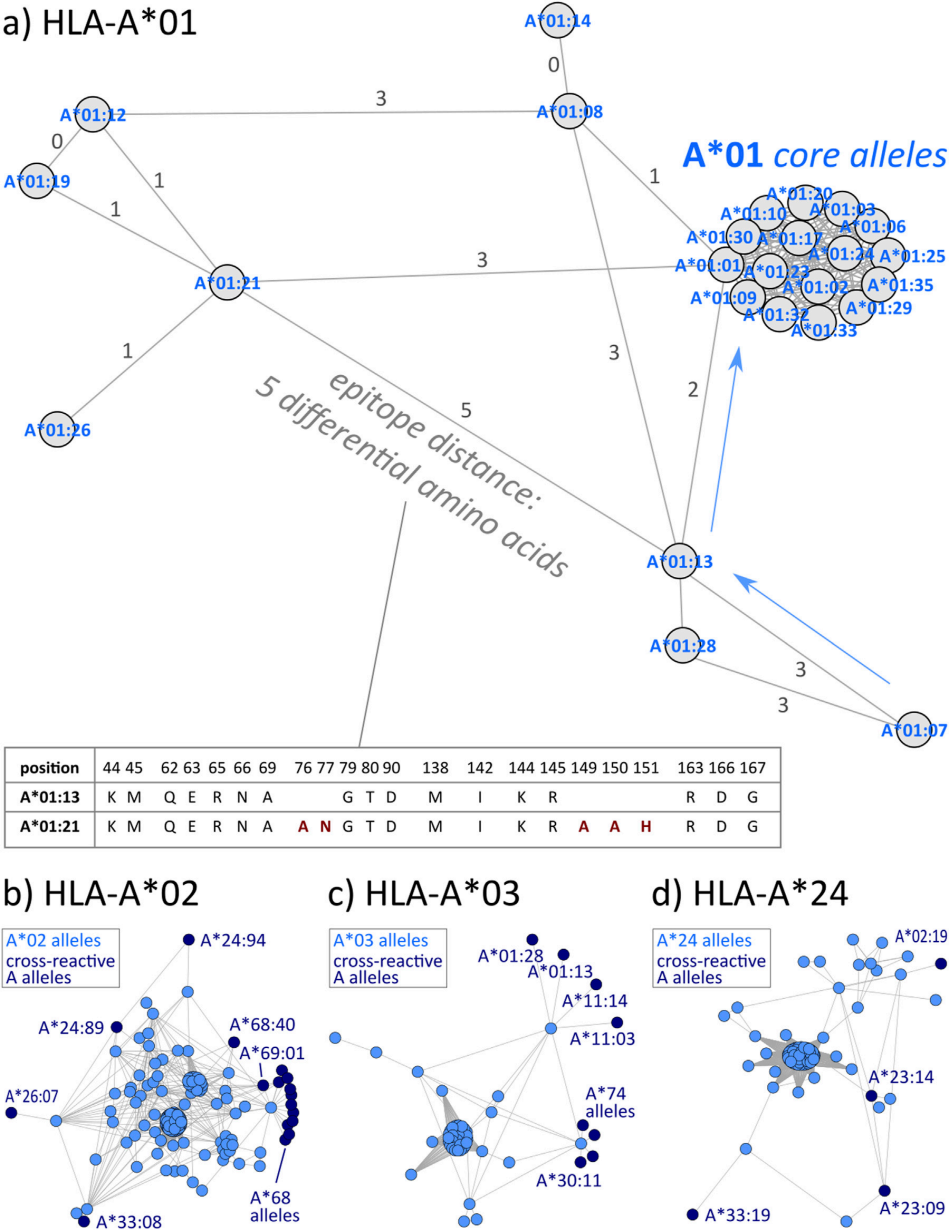
Visualization of the differences of HLA-A alleles towards the other members of the same group. **(A)** The disparity graph shows all HLA-A*01 alleles as nodes, the numbers by the edges rep-resent Hamming distance between the corresponding amino acid residues. If no number is shown, an edge connects monomorphic alleles, i.e., alleles with identical amino acid residues of the corresponding eplets. The node cluster in the upper right part of the graph collects a set of 16 monomorphic alleles. On the other hand, HLA-A*01:07 show a difference of three amino acid residues to monomorphic alleles HLA-A*01:13 and HLA-A*01:28, which, in turn, are two amino acids distant from the main allele group cluster (see labels in the figure). **(B–D)** Corresponding dis-parity graphs for HLA-A*02, A*03, and A*24, respectively. Nodes are colored to distinguish be-tween original and cross-reactive alleles. Edge labels are omitted for clearance.

Next, we intend to visualize differences between the alleles of the individual loci with resolution on single alleles. We therefore count the pairwise differences in polymorphic amino acid residues between each two alleles and translate this distance matrix into a disparity graph structure (Figure 2A). As an example, HLA-A*01:21 differs in 5 positions from the HLA-A*01:13 as highlighted in the excerpt of the corresponding sequences in the figure. HLA-A*01:07 is in 3 amino acid positions different from HLA-A*01:13, which, in turn, differs in 2 positions from the cluster of monomorphic HLA-A*01 “core alleles” (see arrows in the figure).

In the same way, we visualized all HLA-A allele groups (see Figures 2B–D; Supplementary File S2 for interactive disparity graphs of all alleles). Generally, most of these groups show a similar graph structure with one or two clusters of monomorphic alleles, which are surrounded by relatively similar alleles with approximately up to ten polymorphic positions in difference. Except for A*01, all HLA-A groups show similarity into other allele groups. For example, HLA-A*02 alleles form a central cluster of monomorphic alleles surrounded by a “halo” of resembling alleles. Some of these, in turn, show similarity to alleles of the A*24, A*26, and A*33 groups, and especially to 12 alleles of the A*68 group (Figure 2B). Prevalence of these “cross-reactivities” between allele groups is summarized in Table 1. The latter form the well-known HLA-A2/A68 cross-reactivity group. Intriguing here is the close vicinity of A*24 members to the HLA-A*02 group, and of the group HLA-A*03 to A*11 alleles described earlier (Dahlke and Weiss, 1984).

**TABLE 1:** Overview of cross-reactivity and most frequent eplets of selected common HLA-A allele groups.

HLA-A group	Cross-reactivity		Frequent eplets ^a^
	Frequency ^b^	Other group alleles ^c^	
A*01	-	-	44KM, 62QE, 65RNA, 76ANT, 79GT, 90D, 138MI, 144KR, 163RG, 166DG
A*02	7 (5%)	A*68 (12 alleles); A*24 (2); A*26, A*33, A*69 (1)	62GE, 62GK, 79GT, 107W, 127K, 144TKH, 145KHA, 150AAH
A*03	2 (4%)	A*74 (3); A*01, A*11 (2); A*30 (1)	62QE, 65RNA, 79GT, 138MI, 144KR, 150AAH, 161D
A*11	2 (5%)	A*03 (5)	62QE, 65RNA, 79GT, 90D, 138MI, 144KR, 150AAH, 151AHA, 163RW
A*24	5 (6%)	A*23, A*02 (2); A*33 (1)	62EE, 65GK, 80I, 82LR, 127K, 138MI, 144KR, 150AAH, 166DG
A*25	1 (12%)	A*32 (1)	62RR, 65RNA, 76ESI, 80I, 82LR, 90D, 138MI, 149TAH, 163RW
A*26	6 (17%)	A*66 (7); A*02 (1)	62RR, 65RNA, 76ANT, 79GT, 90D, 138MI, 145RT, 149TAH, 163RW
A*30	15 (60%)	A*74 (4); A*31 (2); A*03 (1)	56R, 62QE, 65RNA, 79GT, 138MI
A*32	2 (11%)	A*31 (2)	62QE, 65RNA, 76ESI, 80I, 82LR, 138MI
A*68	2 (4%)	A*02 (3)	62RR, 65RNA, 79GT, 127K, 144TKH, 145KHA, 150AAH

^a^ Eplets confirmed for >80% of the alleles.

^b^ Number and percentage of alleles of the group that are most similar to alleles of another group.

^c^ Cross-reactive alleles from another HLA-A, group; number of affected single alleles of that group is given in parenthesis; alleles are sorted according to decreasing numbers; alleles separated by comma have the same frequency (if no value is given the next value applies).

### 3.3 Disparity graphs of HLA-B and -C

**FIGURE 3:**
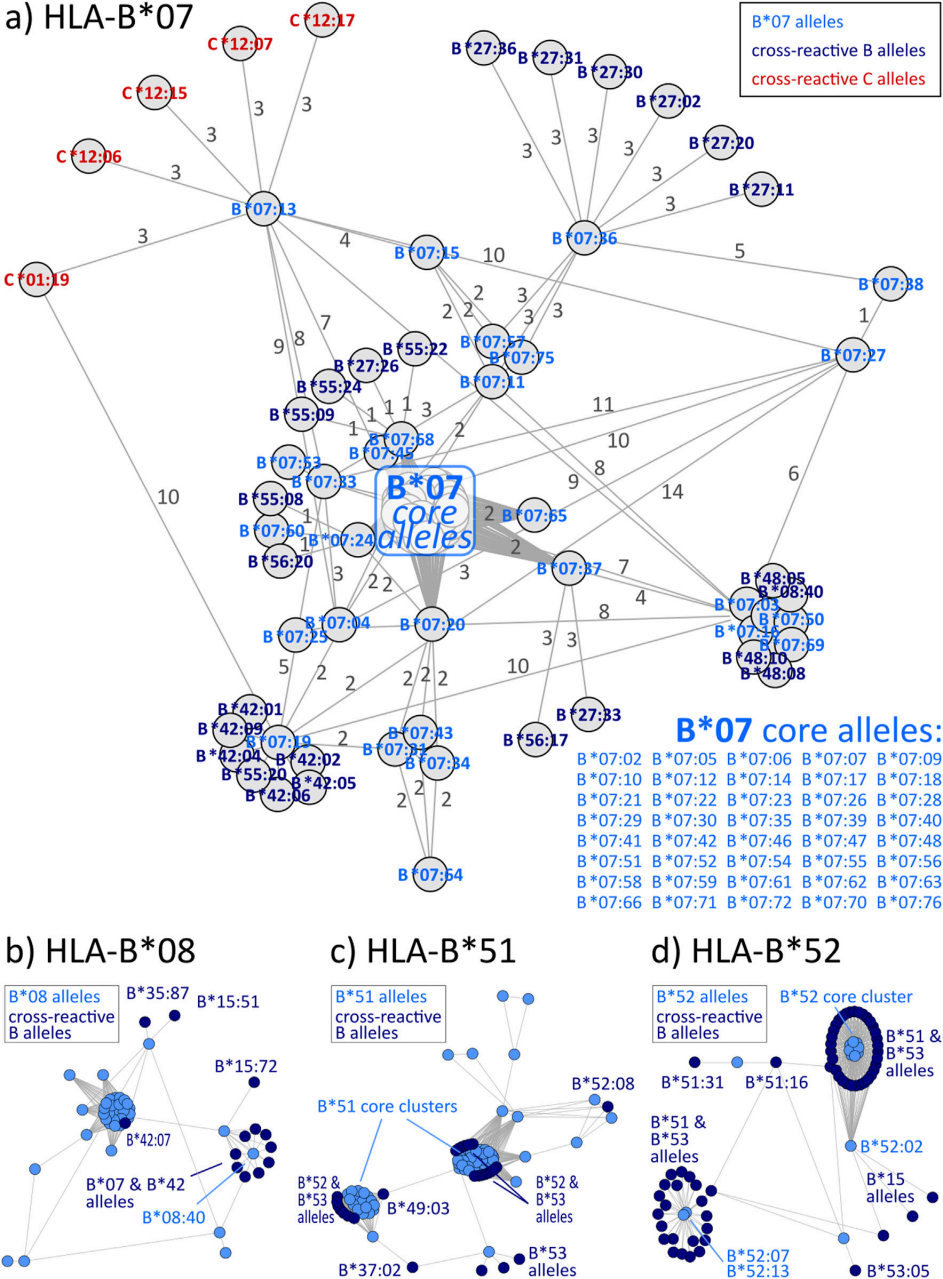
Allele distance graphs of selected HLA-B alleles: **(A)** HLA-B*07, **(B)** HLA-B*08, **(C)** HLA-B*51, and **(D)** HLA-B*52. See description of Figure 2.

Graph visualizations of the amino acid disparities were analogously generated for HLA-B and -C. For B*07, we observe strong interconnection of the group with alleles from other HLA-B groups, especially B*27, B*42, and B*55 (Figure 3A). But also, similarity to C locus alleles can be found for HLA-B*07:13, which is most similar to 4 C*12 alleles and HLA-C*01:19 with distance of three polymorphous amino acids, respectively. Such inter-locus similarities can also be observed for few other HLA-B groups: B*46 has alleles most similar to alleles of the HLA-C*03 group (Zemmour et al., 1992), further pairs are B*67 with C*12 and B*73 with C*07 (see also Table 2; interactive disparity graphs are contained in Supplementary File S3). Overall, HLA-B collects most cross-reactive alleles of all class I loci (see also Table 4). As reported above for the HLA-A group of alleles, we observe here the vicinity of HLA-C*12 to the HLA-B*07 alleles in addition to the reported B*27 and B*54, B*55, B*56 allele groups. Earlier, such cross-reactivity was not reported probably because of the lack of serological reagents defining Cw12 in the past. The “R” group comprising the allele groups of B*18, B*51, B*52, B*53 is also presented (Schwartz et al., 1980). Astonishing is the occurrence of B*37 nearby some of these alleles.

**TABLE 2:** Overview of cross-reactivity and most frequent eplets of selected common HLA-B allele groups.

HLA-B group	Cross-reactivity		Frequent eplets ^a^
	Frequency ^b^	Other group alleles ^c^	
B*07	12 (17%)	B*27 (8 alleles); B*42 (6); B*55 (5); B*48 (3); B*56 (2); B*08 (1); C*12 (4); C*01 (1)	65QIA, 69AA, 70IAQ, 76ESN, 80N, 163EW, 180E
B*08	30 (85%)	B*07 (6); B*48 (3); B*15 (2); B*35, B*42 (1)	69TNT, 71TTS, 76ESN, 80N, 156DA, 180E
B*13	1 (5%)	B*15 (2); B*35 (1)	41T, 44RMA, 69TNT, 80TLR, 82LR, 131S, 144QL, 163EW
B*15	49 (33%)	B*35 (47); B*39 (41); B*57 (13); B*14 (9); B*40, B*78 (4); B*27 (3); B*08, B*48, B*50, B*56 (2); B*37, B*53, B*55, B*59 (1)	69TNT, 71TTS, 76ESN, 80N, 131S, 163LW
B*18	30 (100%)	B*37 (6); B*35 (5); B*53 (3); B*38 (2); B*51 (1)	44RT, 69TNT, 71TTS, 76ESN, 80N, 131S
B*27	8 (17%)	B*15 (5); B*55 (3); B*07 (2); B*54, B*56 (1)	65QIA, 69AA, 82LR, 131S, 163EW
B*35	85 (90%)	B*15 (67); B*18 (28); B*78 (6); B*08, B*39, B*48, B*56 (1)	44RT, 69TNT, 71TTS, 76ESN, 80N, 131S, 163LW
B*40	13 (14%)	B*48 (10); B*15 (5); B*47 (4); B*44, B*50 (2)	41T, 45KE, 69TNT, 71TTS, 76ESN, 80N, 163EW
B*44	8 (13%)	B*45 (6); B*47 (3); B*51 (2); B*37, B*40, B*49, B*50 (1)	41T, 45KE, 69TNT, 80TLR, 82LR, 131S, 163LS/G
B*51	54 (92%)	B*53 (13); B*52 (12); B*37, B*49 (1)	44RT, 69TNT, 80I, 82LR, 131S, 163LW

^a^ Eplets confirmed for >80% of the alleles.

^b^ Number and percentage of alleles of the group that are most similar to alleles of another group.

^c^ Cross-reactive alleles from another HLA-A, group; number of affected single alleles of that group is given in parenthesis; alleles are sorted according to decreasing numbers; alleles separated by comma have the same frequency (if no value is given the next value applies); groups of the HLA-C, locus are highlighted in bold font.

**FIGURE 4:**
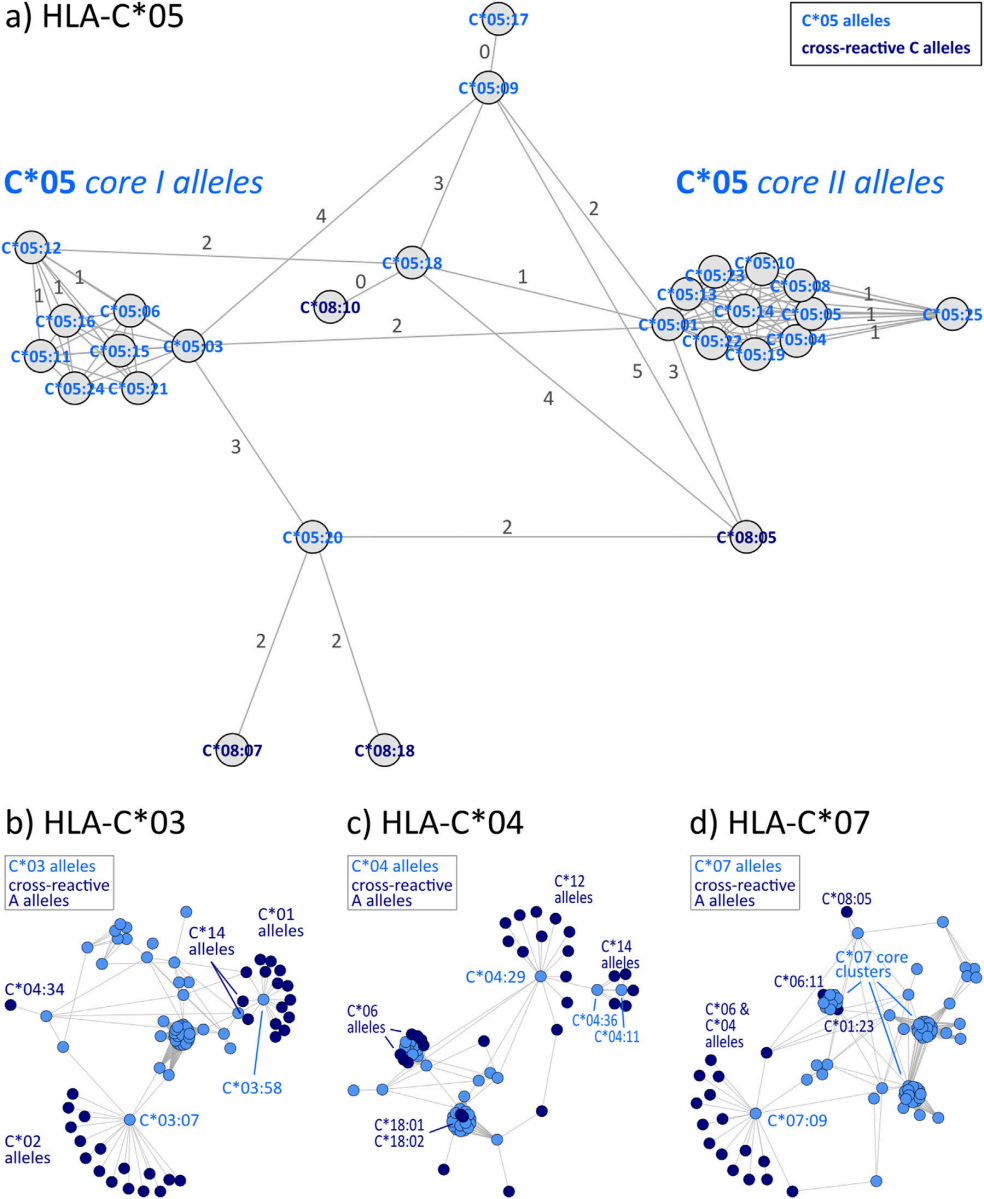
Allele distance graphs of selected HLA-C alleles: **(A)** HLA-C*05, **(B)** HLA-C*03, **(C)** HLA-C*04, and **(D)** HLA-C*07. See description of Figure 2.

HLA-C allele groups partly show close similarity to selected HLA-B alleles as shown above. In general, C allele group disparity graphs show the same structure as those of HLA-A and -B, with “hub” clusters surrounded by more distant allele, which are often linked to alleles of other groups (Figure 4 & Supplementary File S4). In our analyses, C*12 particularly emerges as a linking allele group, which shares similarities towards a variety of other groups such as C*01, C*04, C*06, C*15, and C*16 (Table 3). Other such “intermediary” groups are C*14, C*15, and C*16. The well-known cross-reactivities within the allele groups, e.g., C*05 and C*08, C*04, and C*06 are readily observed. In addition, we observe yet unreported cross-reactivities of C*14, C*17 and C*18 allele groups with the ones reported above.

**TABLE 3:** Overview of cross-reactivity and most frequent eplets of selected common HLA-C allele groups.

HLA-C group	Cross-reactivity		Frequent eplets ^a^
	Frequency ^b^	Other group alleles ^c^	
C*01	11 (4%)	C*12 (15); C*07 (12); C*16 (7); C*14 (6); C*04 (3); C*15 (2); C*06, C*08 (1)	65QKR, 73TVS, 76VRN, 80N
C*02	1 (5%)	C*03 (2)	21H, 65QKR, 80K, 163EW
C*03	4 (8%)	C*02 (16); C*01 (13); C*14 (2); C*04 (1)	21H, 65QKR, 73TVS, 76VRN, 80N, 163LW, 173K
C*04	31 (89%)	C*12 (12); C*06 (7); C*14 (5); C*01, C*18 (3)	65QKR, 73AN, 80K, 90D
C*05	2 (9%)	C*08 (4)	65QKR, 80K, 138K, 177KT
C*06	11 (55%)	C*07 (9); C*04 (8); C*12, C*16 (2); C*01, C*18 81)	65QKR, 73AN, 80K, 90D
C*07	12 (18%)	C*04, C*06 (8); C*01, C*08, C*15 (1)	76VRN, 80N, 90D
C*12	20 (100%)	C*06 (9); C*15 (5); C*01, C*07 (3); C*05, C*16 (2); C*04 (1)	65QKR, 80N
C*15	3 (17%)	C*12 (2); C*01 (1)	21H, 80K, 193PV
C*16	10 (100%)	C*01 (4); C*12 (2); C*14 (1)	65QKR

^a^ Eplets confirmed for >80% of the alleles.

^b^ Number and percentage of alleles of the group that are most similar to alleles of another group.

^c^ Cross-reactive alleles from another HLA-A, group; number of affected single alleles of that group is given in parenthesis; alleles are sorted according to decreasing numbers; alleles separated by comma have the same frequency (if no value is given the next value applies).

### 3.4 Eplet disparity of HLA-class II allele groups

**FIGURE 5:**
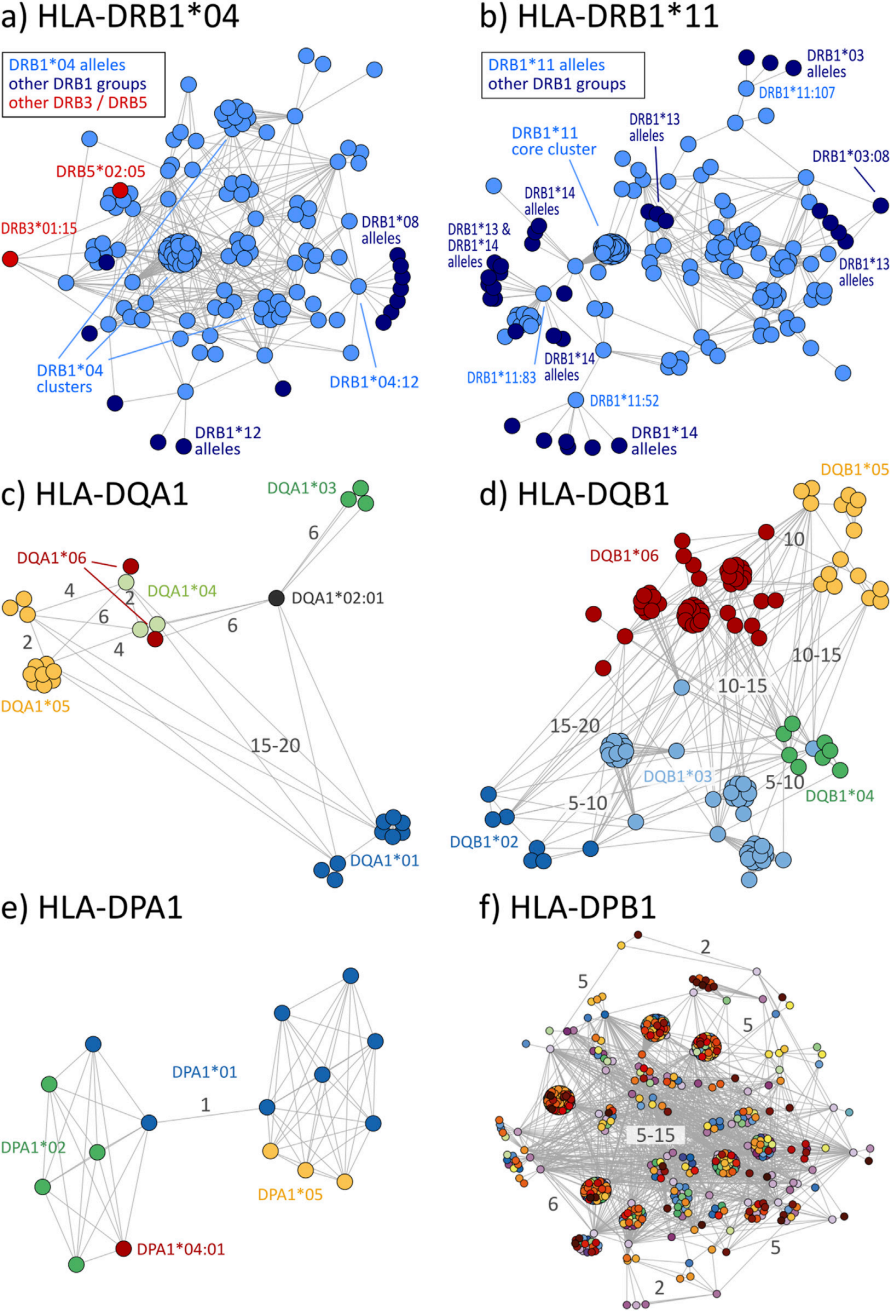
Disparity graphs of HLA-class II loci: **(A)** HLA-DRB1*04, **(B)** HLA-DRB1*11, **(C)** HLA-DQA1, **(D)** HLA-DQB1, **(E)** HLA-DPA1, and **(F)** HLA-DPB1. For DQ and DP, all allele groups are shown in integrated graphs. See also description of Figure 2.

Disparity analysis of HLA-DRB1 allele groups show similar pattern to class I graphs (Figures 5A, B; interactive disparity graphs of class II allele groups are provided as Supplementary File S5) and similar prevalence of cross-reactivity (Table 4). We observe highly interconnected disparity graphs for these allele groups with multiple clusters of monomorphic alleles. Thereby, cross-reactivity towards other DRB1 groups is less common than in HLA-B and -C. About 15% of DRB1 alleles reveal cross-reactivity to other groups, which is comparable to the HLA-A allele groups (see Table 4). DRB3, 4, and 5 comprise only few allele groups, whereby DRB3 and 5 groups show frequent cross-reactivity (Table 4) especially to DRB1*04 and DRB1*16 alleles (Supplementary File S5).

**TABLE 4:** Prevalence of cross-reactivity in HLA-class I and II loci.

Locus	# Groups	# Alleles	Avg. # Alleles per group	Cross-reactive alleles (#/%)
A	21	586	28	70/12
B	36	978	27	516/53
C	14	331	24	120/36
DRB1	13	858	66	131/15
DRB3	3	46	15	24/52
DRB4	1	8	8	0/0
DRB5	2	17	9	13/76
DQA1	6	29	5	6/21
DQB1	5	125	25	7/6
DPA1	4	16	4	16/100
DPB1	653	655	1	655/100

Allele groups of DQ and DP loci were jointly collected in disparity graphs, respectively, due to the very few groups in these loci (similar locus-wise disparity graphs for HLA-A, B, C, and DRB1 are provided as Supplementary File S6). We found that DQA1 and DQB1 form a clear cluster structure of their groups (Figures 5C, D). These clusters are, in turn, connected with a position distance of about 5–20 polymorphic amino acids. The group-wise disparity graphs show that DQA1 and DQB1 are relatively homogeneous with cross-reactivity of only 6%–21% of the alleles (Table 4 and Supplementary File S5).

For the allele groups of the DP locus, our analysis shows that all alleles of DPA1 are virtually monomorphic (Figure 5E). In contrast, the more than 600 individual alleles of DPB1 distribute over more than 600 allele groups with strong inter-connectivity among them (Figure 5F). Here, cross-reactivity of 100% of all DPB1 alleles is trivial, underlining the special structure of DPB1 group nomenclature.

## 4 Discussion

The HLA class I antigens and disparities of their allele groups can be visualized as shown in the present report. Our approach allows to evaluate similarities and disparities between the alleles of an allele group. In the early days of HLA typing, the cross-reactive groups were used to show the similarity of serological specificities to each other (Rodey and Fuller, 1987). However, their value in solid organ transplantation is disputed. While in the US in some instances they are included in the matching procedure, in mainland Europe reports show that they have an adverse effect (Stobbe et al., 2000a; Stobbe et al., 2000b). Meanwhile, eplet mismatches are on the way to be considered in the allocation procedure in Canada (Mohammadhassanzadeh et al., 2021).

We based our approach on already confirmed eplets to generate most reliable disparity graphs, but which also limits our study in terms of potentially missing differential and/or shared eplets. Moreover, EpRegistry will continue to evolve like any nomenclature database and additional eplets will be defined and antibody-confirmed in the future. We therefore see our study as a proof of concept, which could be updated upon public release of new data and potentially extended to all eplets contained in the data base. Whether our findings will allow in some instances to help patients at need of a suitable graft remains still open until reliable data are available. Yet it is a potential alternative to use cross-reactive mismatched alleles for patients without proper donor because of the very low number of incompatibilities in terms of eplets and epitopes. Following the general understanding that the more incompatibilities are worse for the patient (Wiebe and Nickerson, 2018), we propose an adjustment: The less incompatibilities the better for the patient (Schawalder et al., 2021; Tambur and Das, 2023).

The data and graphs presented here makes also clear that the current molecular-based nomenclature does not reflect the biological one when immunological responses are in the focus. We do not opt for a radical change of the currently used nomenclature, but we advocate the use of a more clinic-oriented addendum in which similar alleles are grouped to offer to the fellow clinicians a better understand of the topic and to a better treatment to the patients. Mismatches with immunological similarities could be treated with lower doses of immunosuppressant than higher disparities. This shall be discussed between the different faculties involved in transplantation.

In practice, alleles with the same amino acid sequence in the respective positions could be considered as equally recognizable by the immune system. Earlier, organ allocation organizations such as Eurotransplant, considered some antigens as being “logical identical”, for example, DR1 and DR103, or in molecular terms DRB1*01:01 and DRB1*01:03. Their definition at that time was done serologically. The data presented here were obtained by the two state-of-the-art methods next-generation sequencing (NGS) and real-time polymerase chain reaction. It could be used by researchers and organ allocation organization to redefine “identical” alleles and acceptable mismatches. Surely, we need clinical data sets to verify that such different alleles do not provoke an immune response in the set up proposed here as, for example, shown for DRB1*14:01 and DRB1*14:54 (Pasi et al., 2011). We could envisage an introduction of functional matching especially for the HLA alleles provoking a humoral response. That means that alleles bearing the same eplet and therefore - according to current knowledge - not provoking an antibody production could be regarded as compatible. In the case of “allele-specific” antibodies, which are mainly due to a not shared epitope between the two alleles, should be avoided due to epitope incompatibility. Our findings (Lehmann et al., 2023; Lehmann et al. manuscript in preparation) clearly show that repeated eplet mismatches can be deleterious and should be also avoided.

Selected donor-recipient-pairs of a cohort of kidney transplants (HLA, 2024a) are summarized in terms of the number of HLA-class I allele mismatches and the corresponding eplet (Table 5). This data supports the key finding of our allele disparity analyses: It shows that some single allele mismatches imply only one or very few mismatched eplets (rows 1, 4 and 7 in Table 5), but a single allele mismatch can also lead to a multitude of up to eight eplet mismatches (rows 2, 5, and 8). Even more, mismatch of both alleles of a locus sometimes leads to fewer mismatched eplets compared to other single allele mismatches (rows 3, 6, and 9).

**TABLE 5:** HLA-class I characteristics of selected donor-recipient pairs from a living kidney transplant cohort. Each row represents an individual pair with varying HLA-A, -B, and -C allele and eplet discrepancies, respectively.

Id	Recipient	Donor	Antigen/Allele mismatches	Eplet mismatches	
1	A*02:01/A*03:01	A*02:01/A*30:01	1	1	56R
2	A*02:01/A*68:02	A*01:01/A*02:01	1	8	44KM, 62QE, 76ANT, 138MI, 144KR, 163R, 163RG, 166DG
3	A*02:05/A*32:01	A*03:01/A*24:02	2	5	62EE, 65GK, 144KR, 161D, 166DG
4	B*35:03/B*44:02	B*35:03/B*39:01	1	1	158T
5	B*35:01/B*56:01	B*35:01/B*44:02	1	6	41T, 45KE, 80TLR, 82LR, 156DA, 163LS/G
6	B*07:02/B*35:02	B*15:01/B*44:02	2	6	41T, 44RMA, 45KE, 80TLR, 156DA, 163LS/G
7	C*04:01/C*07:02	C*07:02/C*15:02	1	1	21H
8	C*07:01/C*07:04	C*03:04/C*07:01	1	6	21H, 73TVS, 163LW, 173K, 193PV, 219W
9	C*04:01/C*07:02	C*03:04/C*05:01	2	5	21H, 73TVS, 138K, 173K, 177KT

In summary, we find a disparity-driven variability of the number of eplet mismatches in a series of real kidney allocations, revealing the need to further evaluate data of transplant cohorts in this respect and to correlate quantity and quality of eplet mismatches with outcome and graft survival.

## 5 Conclusion

Disparity graphs of polymorphic amino acid sequences of HLA epitopes visualize similarity and dissimilarity patterns in the allele groups. They can be seen as a strictly data-driven reminiscence of the cross-reactivity maps of Darke (1984), Mayr (1988), now functionally based on polymorphic, antibody-confirmed binding sites as represented by the epitopes. Our results show that the historically grown HLA allele nomenclature is inconsistent and motivate their amendment with focus on eplets.

## Data Availability

Publicly available datasets were analyzed in this study. This data can be found here: https://epregistry.com.br/.

## References

[B1] AFND (2020). The allele frequency net database. Available at: https://www.allelefrequencies.net/hla6006a.asp.

[B2] Bezstarosti S. Bakker K. H. Kramer C. S. M. Fijter J. W. de Reinders M. E. J. Mulder A. (2021). A comprehensive evaluation of the antibody-verified status of eplets listed in the HLA epitope registry. Front. Immunol. 12, 800946. 10.3389/fimmu.2021.800946 35154076 PMC8831796

[B3] Csardi G. Nepusz T. (2006). The igraph software package for complex network research. InterJournal 1695. Available at: https://igraph.org.

[B4] Dahlke M. B. Weiss K. L. (1984). Platelet transfusion from donors mismatched for crossreactive HLA antigens. Transfusion 24 (4), 299–302. 10.1046/j.1537-2995.1984.24484275567.x 6464151

[B5] Darke C. (1984). A serological study of the HLA-B17 cross-reactive group. Tissue Antigens 23 (3), 141–150. 10.1111/j.1399-0039.1984.tb00024.x 6203186

[B6] D’Orsogna L. J. Roelen D. L. Doxiadis I. I. N. Claas F. H. J. (2012). TCR cross-reactivity and allorecognition: new insights into the immunogenetics of allorecognition. Immunogenetics 64 (2), 77–85. 10.1007/s00251-011-0590-0 22146829 PMC3253994

[B7] Duquesnoy R. J. (2006). A structurally based approach to determine HLA compatibility at the humoral immune level. Hum. Immunol. 67 (11), 847–862. 10.1016/j.humimm.2006.08.001 17145365 PMC2169290

[B8] HLA (2024a). Abstracts for the joint 37th European immunogenetics and histocompatibility conference, Geneva, Switzerland, may 20 - 23, 2024. HLA 103 (Suppl. 1), 7–144. 10.1111/tan.15475

[B9] HLA (2024b). HLA eplet registry. Available at: https://www.epregistry.com.br (Accessed August 8, 2024).

[B10] MacPherson B. R. (1989). HLA antibody formation within the HLA-A1 crossreactive group in multitransfused platelet recipients. Am. J. Hematol. 30 (4), 228–232. 10.1002/ajh.2830300407 2784623

[B11] Mayr W. R. (1988). HLA cross reactions.

[B12] Meng T. Bezstarosti S. Singh U. Yap M. Scott L. Petrosyan N. (2023). Site-directed mutagenesis of HLA molecules reveals the functional epitope of a human HLA-A1/A36-specific monoclonal antibody. HLA 101 (2), 138–142. 10.1111/tan.14895 36401817 PMC10099858

[B13] Mohammadhassanzadeh H. Oualkacha K. Zhang W. Klement W. Bourdiec A. Lamsatfi J. (2021). On path to informing hierarchy of eplet mismatches as determinants of kidney transplant loss. Kidney Int. Rep. 6 (6), 1567–1579. 10.1016/j.ekir.2021.03.877 34169197 PMC8207474

[B14] Pasi A. Crocchiolo R. Bontempelli M. Carcassi C. Carella G. Crespiatico L. (2011). The conundrum of HLA-DRB1*14:01/*14:54 and HLA-DRB3*02:01/*02:02 mismatches in unrelated hematopoietic SCT. Bone Marrow Transpl. 46 (7), 916–922. 10.1038/bmt.2010.246 20972469

[B15] Rodey G. E. Fuller T. C. (1987). Public epitopes and the antigenic structure of the HLA molecules. Crit. Rev. Immunol. 7 (3), 229–267. 2441930

[B16] Schawalder L. Hönger G. Kleiser M. van Heck M. R. van de Pasch L. A. L. Vendelbosch S. (2021). Development of an immunogenicity score for HLA-DQ eplets: a conceptual study. HLA 97 (1), 30–43. 10.1111/tan.14110 33068062 PMC7756751

[B17] Schwartz B. D. Luehrman L. K. Lee J. Rodey G. E. (1980). A public antigenic determinant in the HLA-B5 cross-reacting group--a basis for cross-reactivity and a possible link with Behcet's disease. Hum. Immunol. 1 (1), 37–54. 10.1016/0198-8859(80)90008-7 6167541

[B18] Stobbe I. van der Meer-Prins E. M. Lange P. de Oudshoorn M. Doxiadis I. I. Claas F. H. (2000a). *In vitro* CTL precursor frequencies do not reflect a beneficial effect of cross-reactive group (CREG) matching. Hum. Immunol. 61 (9), 879–883. 10.1016/s0198-8859(00)00160-9 11053631

[B19] Stobbe I. van der Meer-Prins E. M. Lange P. de Oudshoorn M. Meester J. de Doxiadis I. I. (2000b). Cross-reactive group matching does not lead to a better allocation and survival of donor kidneys. Transplantation 70 (1), 157–161. 10919594

[B20] Tambur A. R. Das R. (2023). Can we use eplets (or molecular) mismatch load analysis to improve organ allocation? The hope and the hype. Transplantation 107 (3), 605–615. 10.1097/TP.0000000000004307 36163639 PMC9944744

[B21] Thorsby E. (2009). A short history of HLA. Tissue Antigens 74 (2), 101–116. 10.1111/j.1399-0039.2009.01291.x 19523022

[B22] van Heemst J. Jansen DTSL Polydorides S. Moustakas A. K. Bax M. Feitsma A. L. (2015). Crossreactivity to vinculin and microbes provides a molecular basis for HLA-based protection against rheumatoid arthritis. Nat. Commun. 6, 6681. 10.1038/ncomms7681 25942574

[B23] Vittoraki A. G. Fylaktou A. Tarassi K. Tsinaris Z. Siorenta A. Petasis G. C. (2021). Hidden patterns of anti-HLA class I alloreactivity revealed through machine learning. Front. Immunol. 12, 670956. 10.3389/fimmu.2021.670956 34386000 PMC8353326

[B24] Wiebe C. Nickerson P. (2018). Human leukocyte antigen mismatch and precision medicine in transplantation. Curr. Opin. Organ Transpl. 23 (4), 500–505. 10.1097/MOT.0000000000000540 29787417

[B25] Zemmour J. Gumperz J. E. Hildebrand W. H. Ward F. E. Marsh S. G. Williams R. C. (1992). The molecular basis for reactivity of anti-Cw1 and anti-Cw3 alloantisera with HLA-B46 haplotypes. Tissue Antigens 39 (5), 249–257. 10.1111/j.1399-0039.1992.tb01943.x 1384166

