# Supplementary figures and images for: epiArt: a graphical HLA eplet amino acid repertoire translation reveals the need for an epitope driven revision of allele group nomenclature
Source: Front Genet. 2024 Oct 16;15:1449301. doi: 10.3389/fgene.2024.1449301 (PMC11521843; doi:10.3389/fgene.2024.1449301)

a) HLA-A

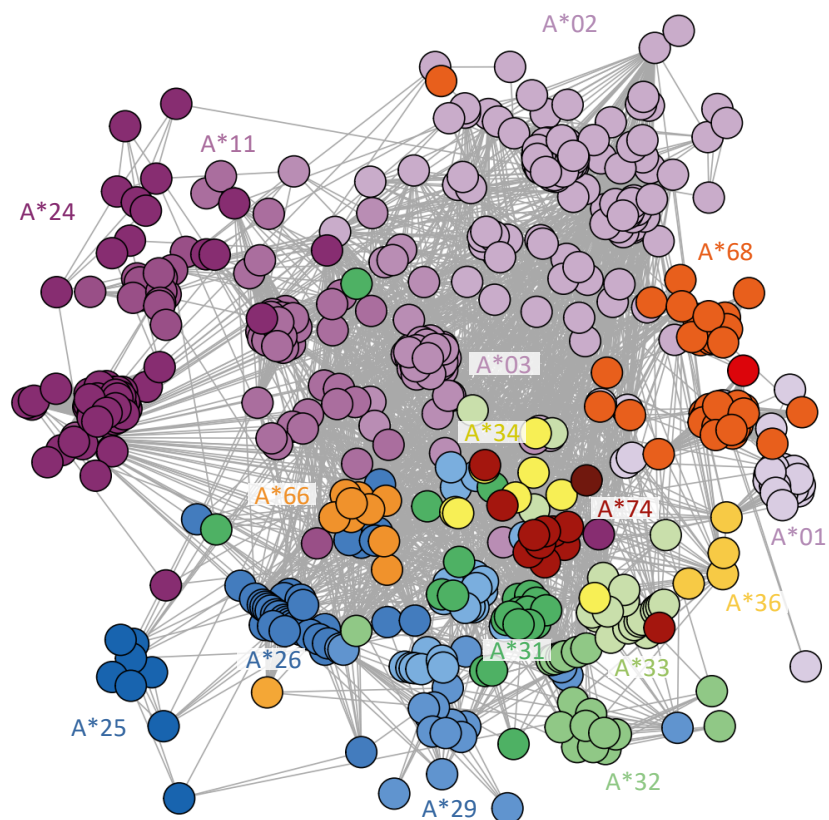

b) HLA-B

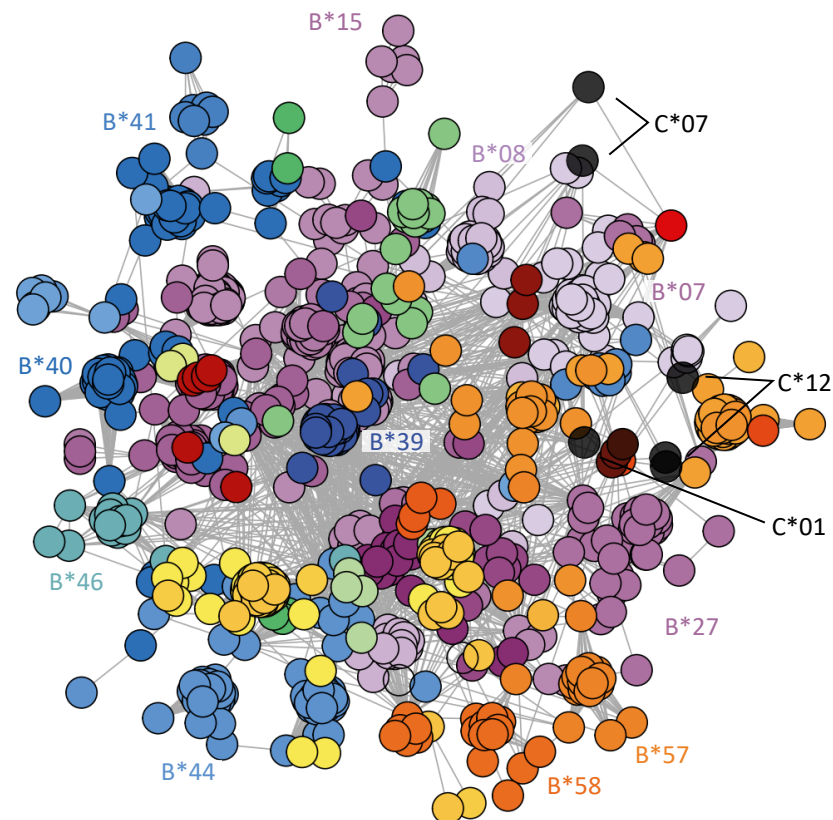

c) HLA-C

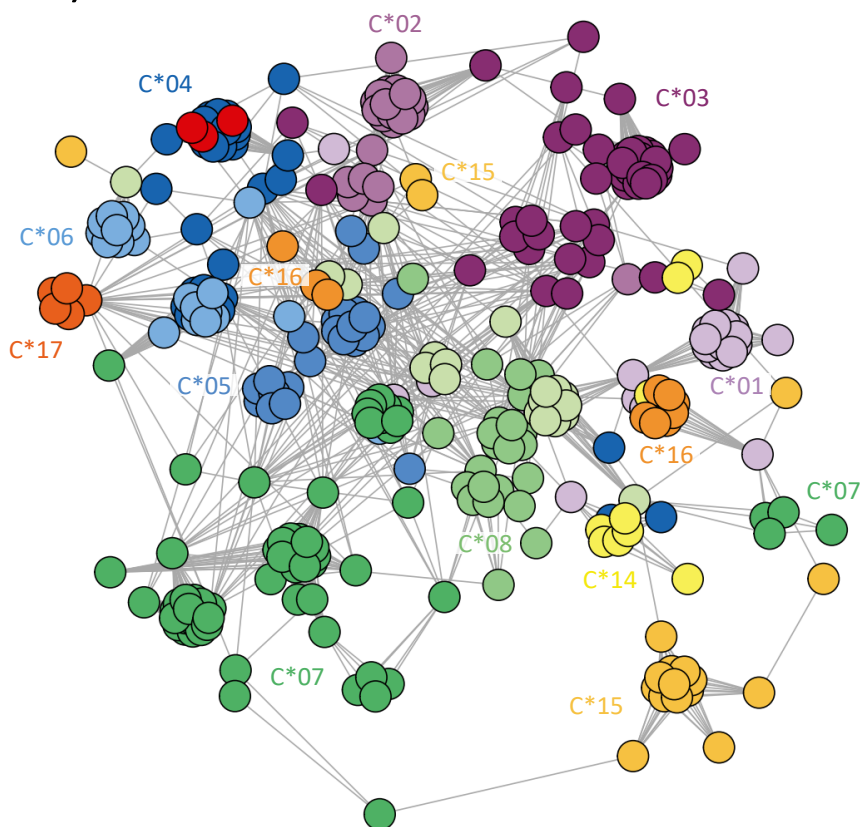

d) HLA-DRB1

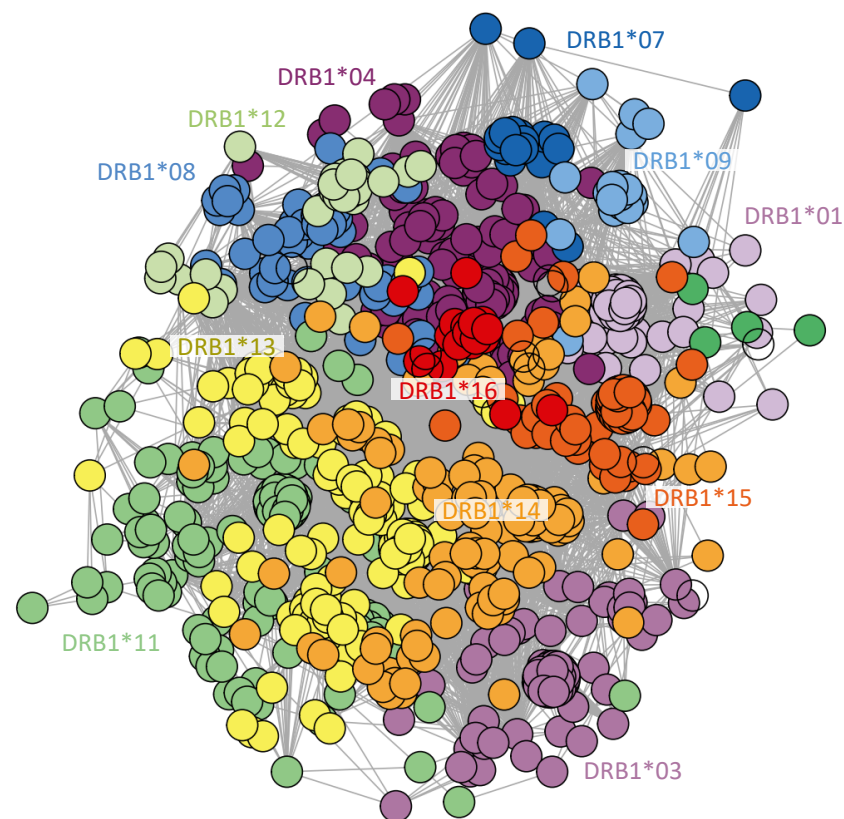

Supplement: Supplementary file 1 [file DataSheet3.PDF]

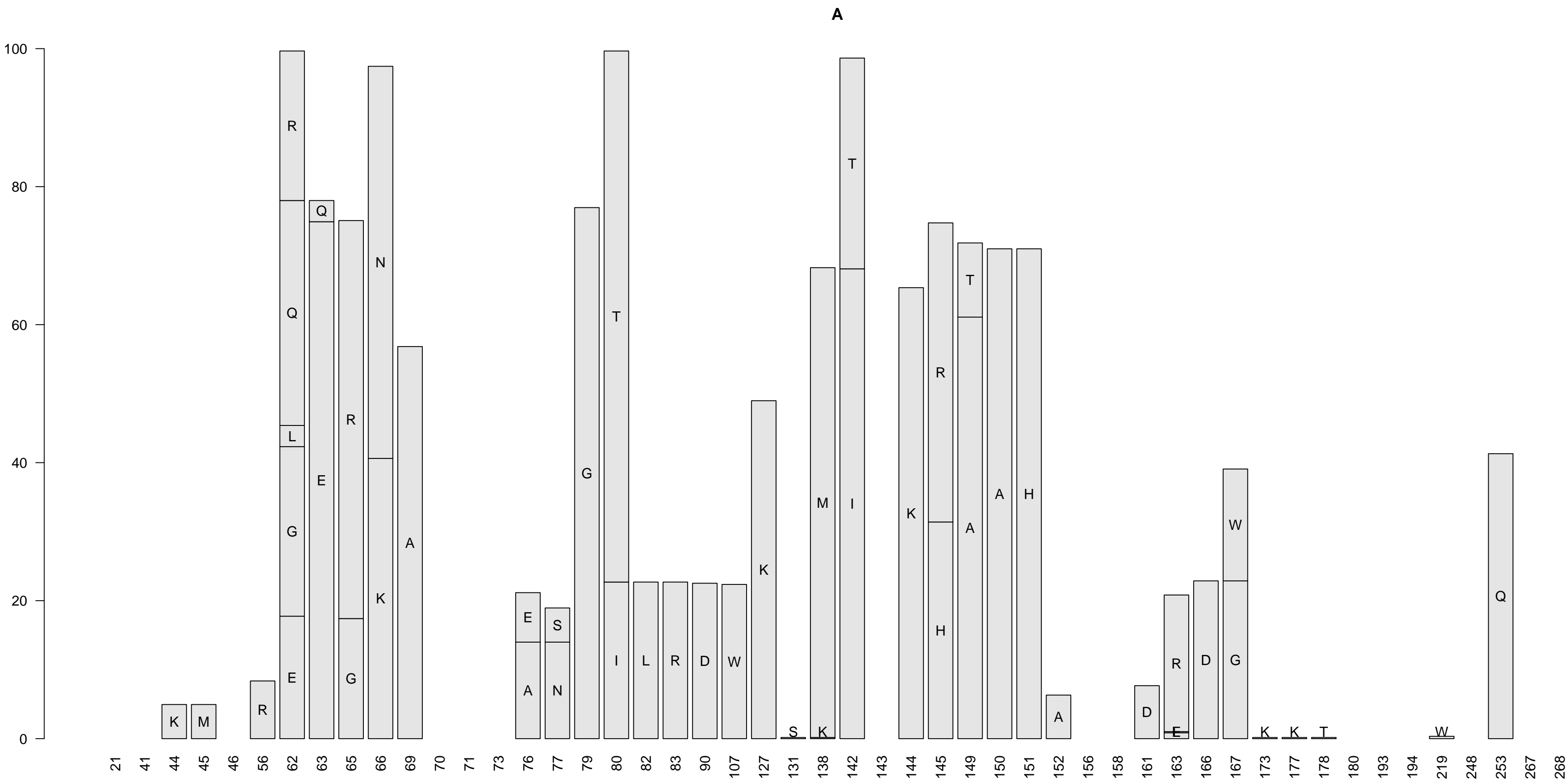

B

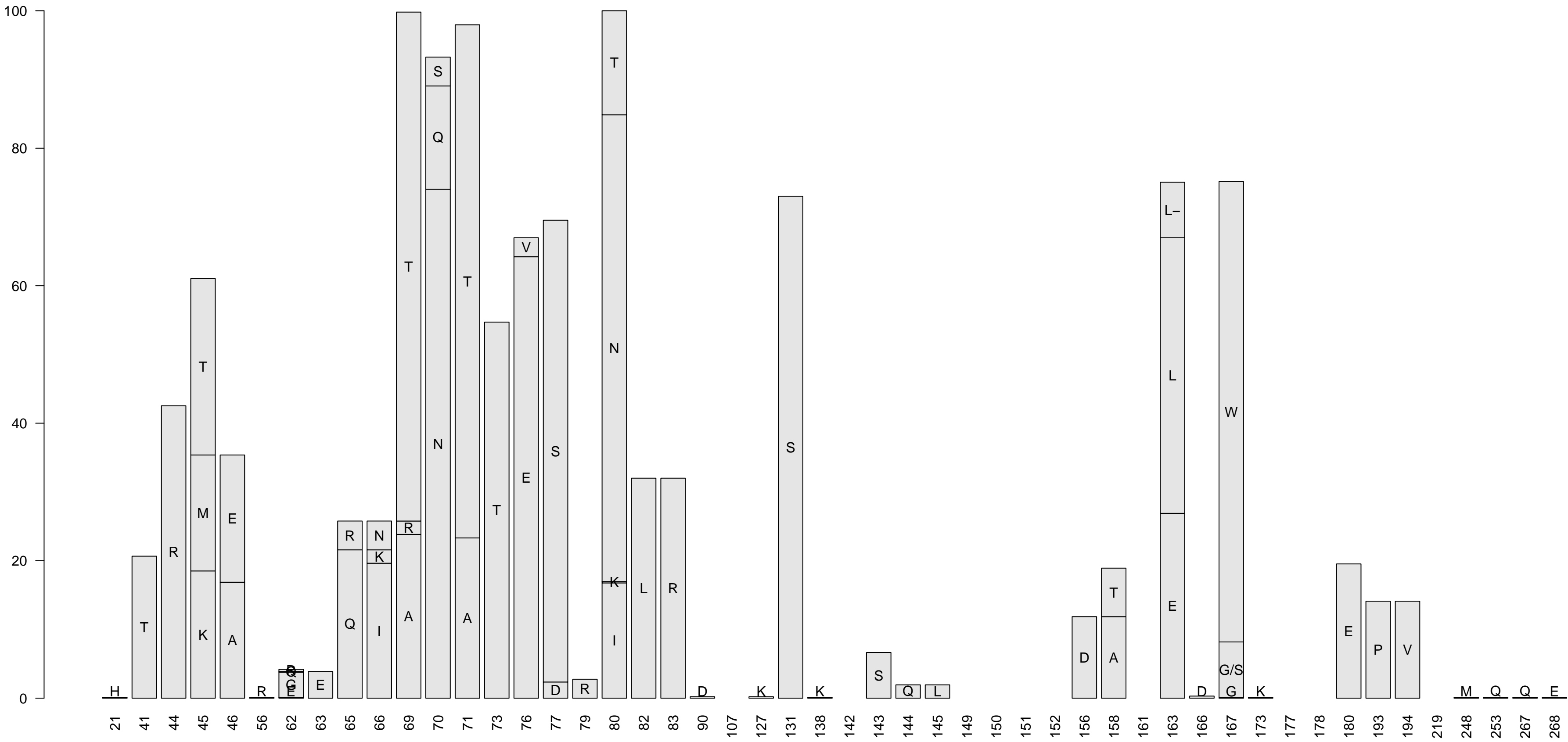

C

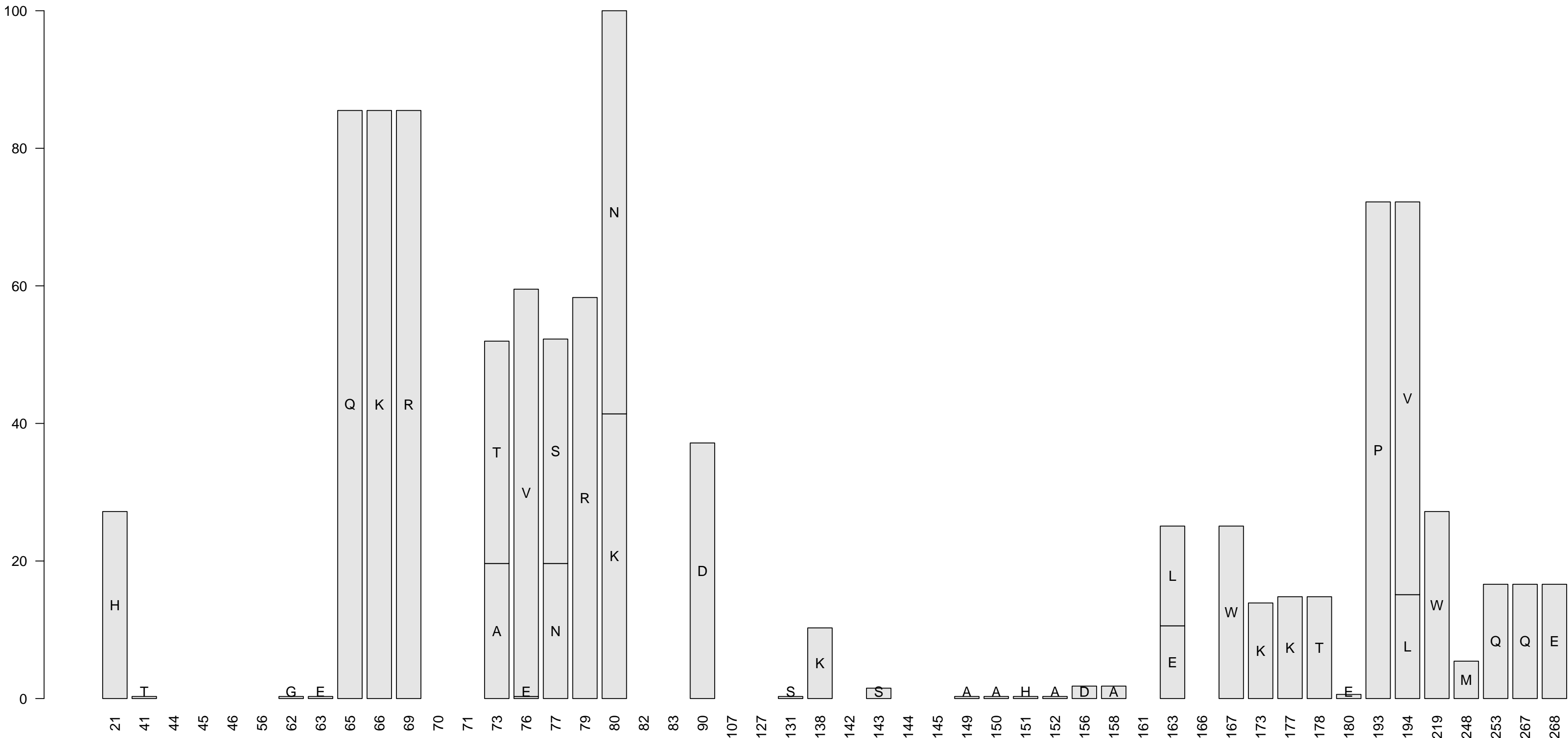

DPA1

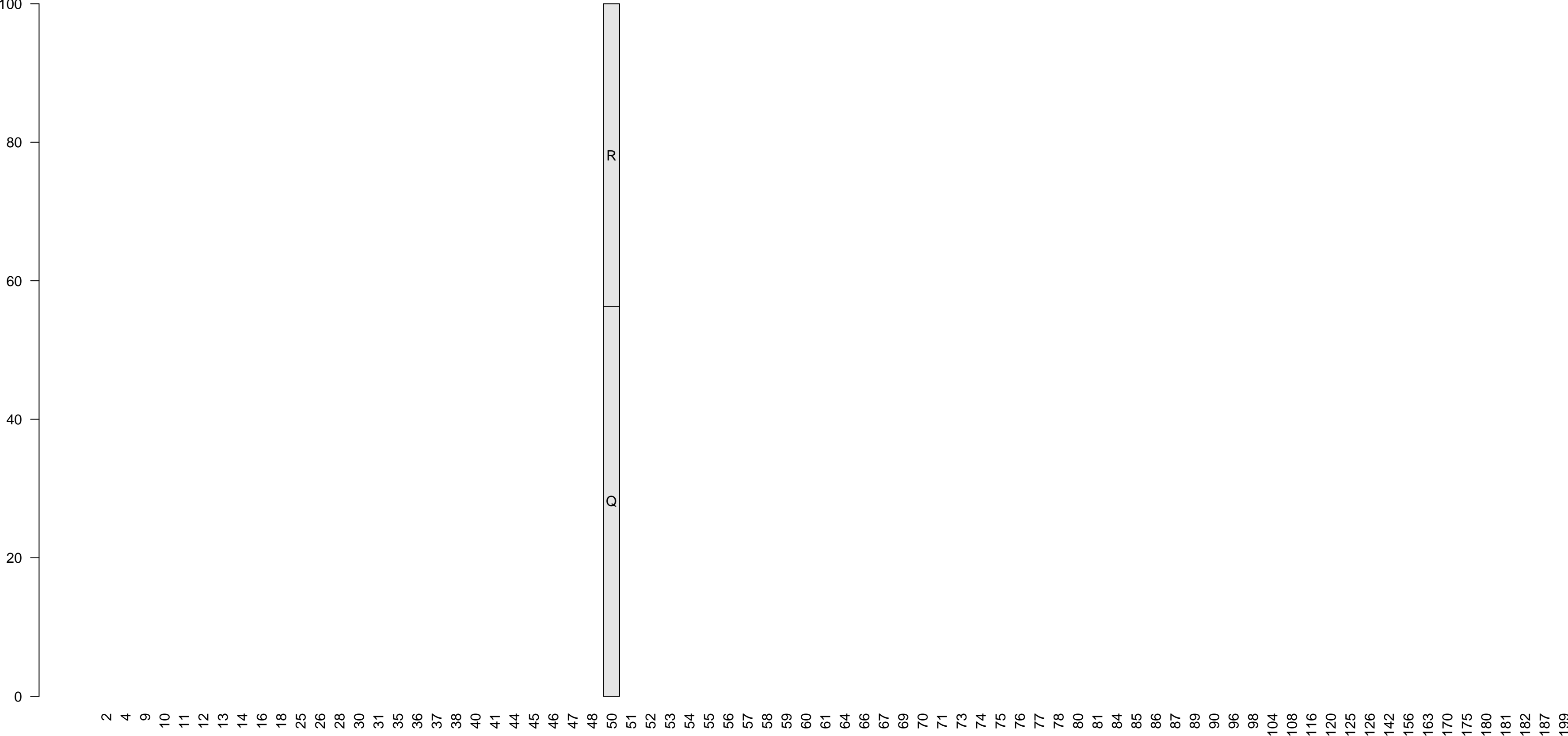

DPB1

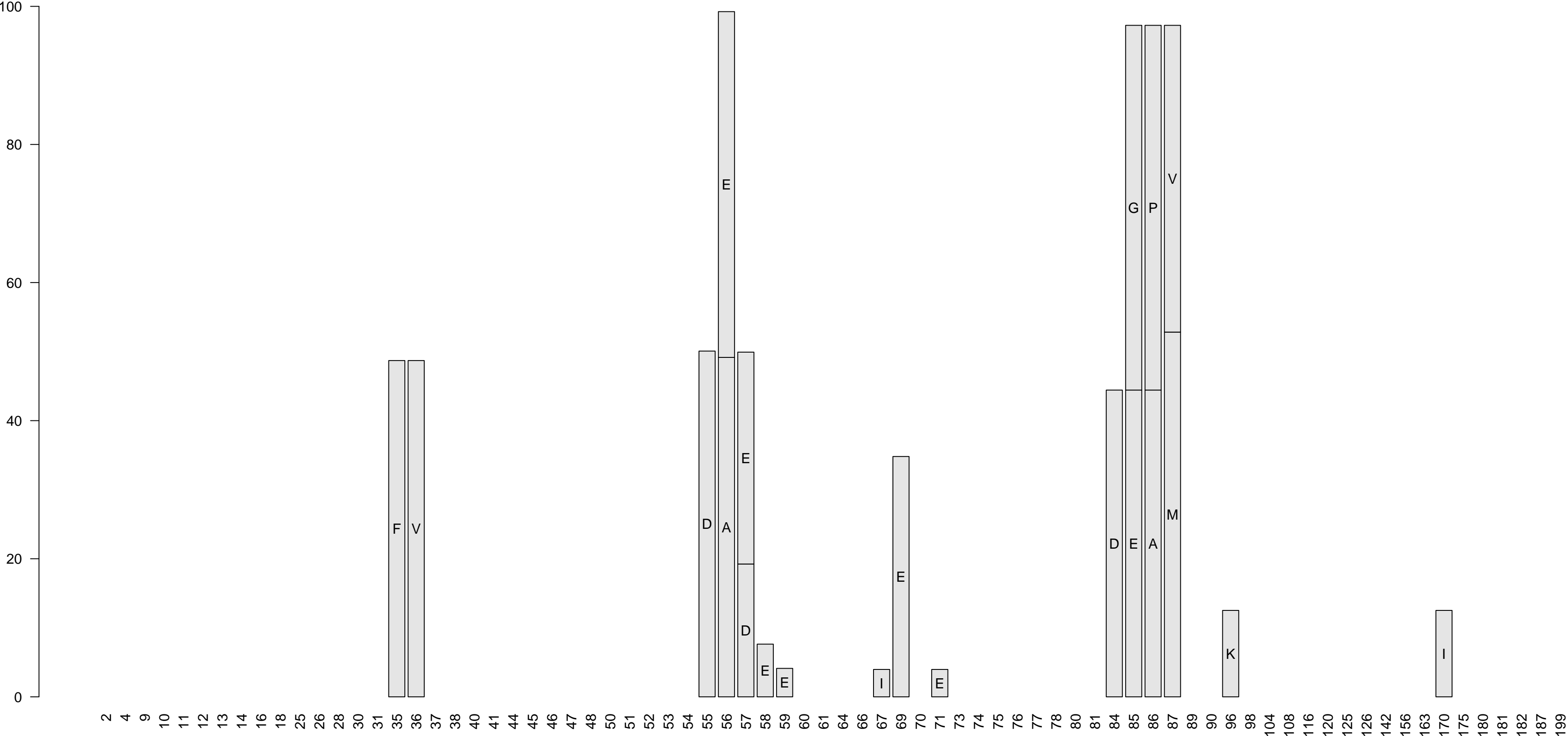

DQA1

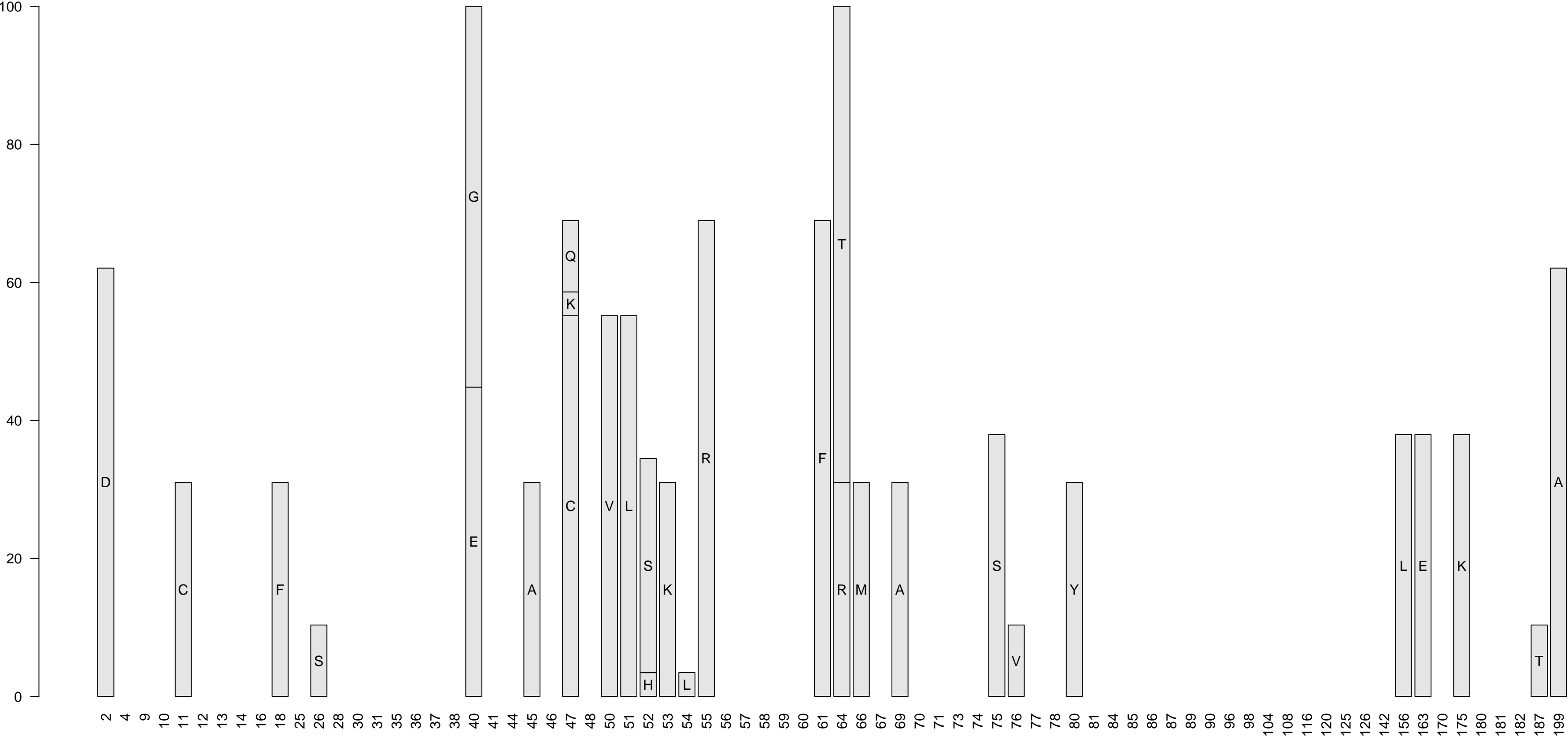

DQB1

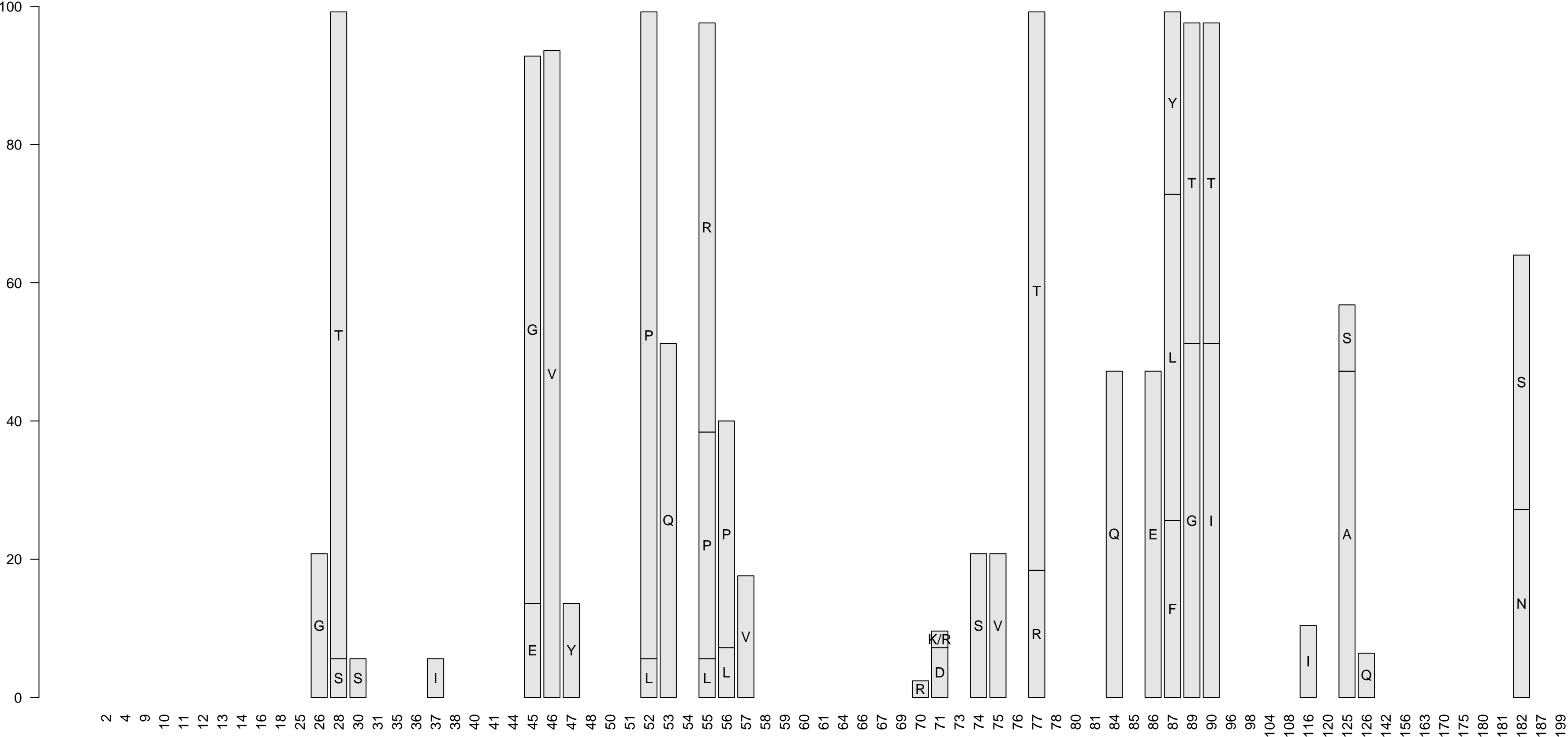

DRB1

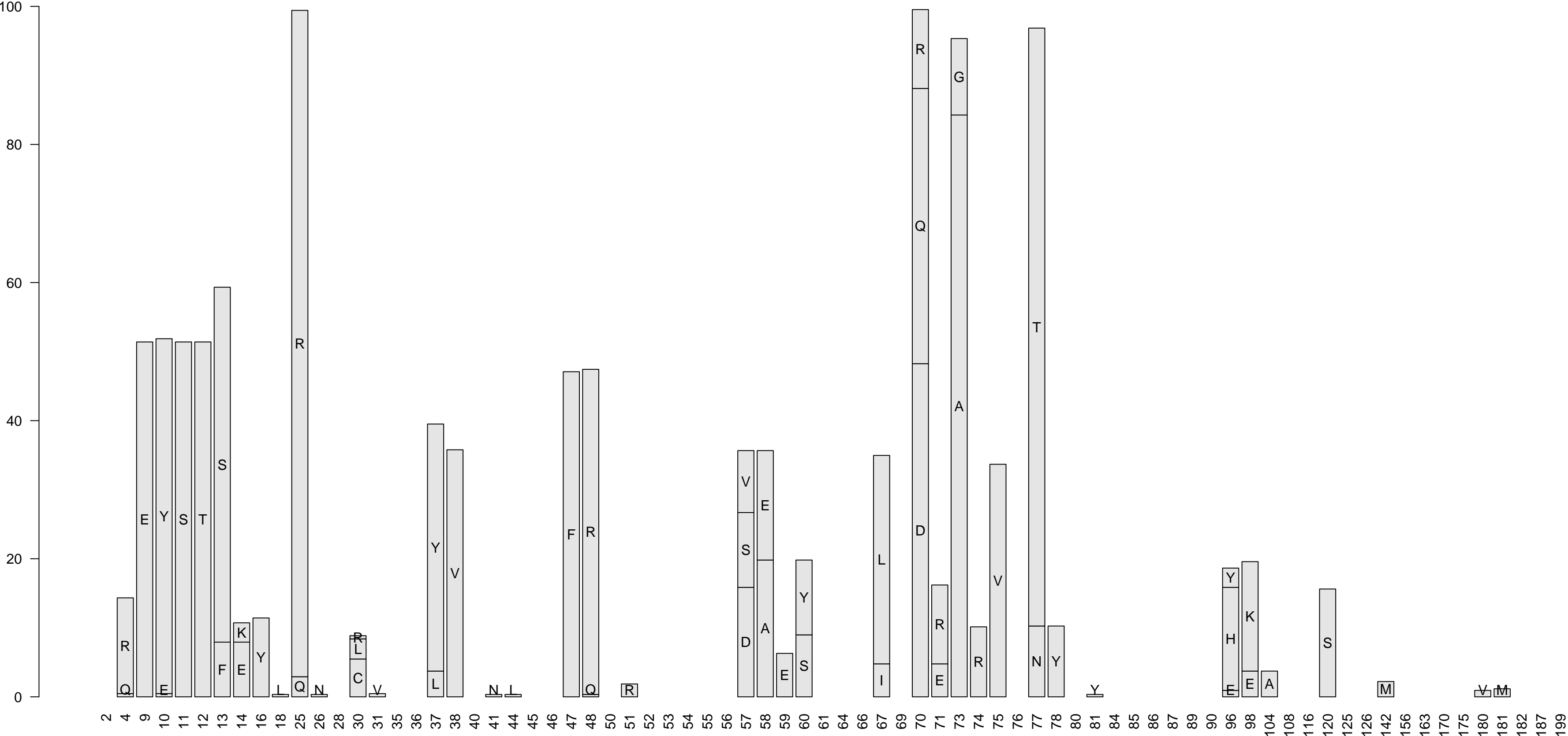

DRB3

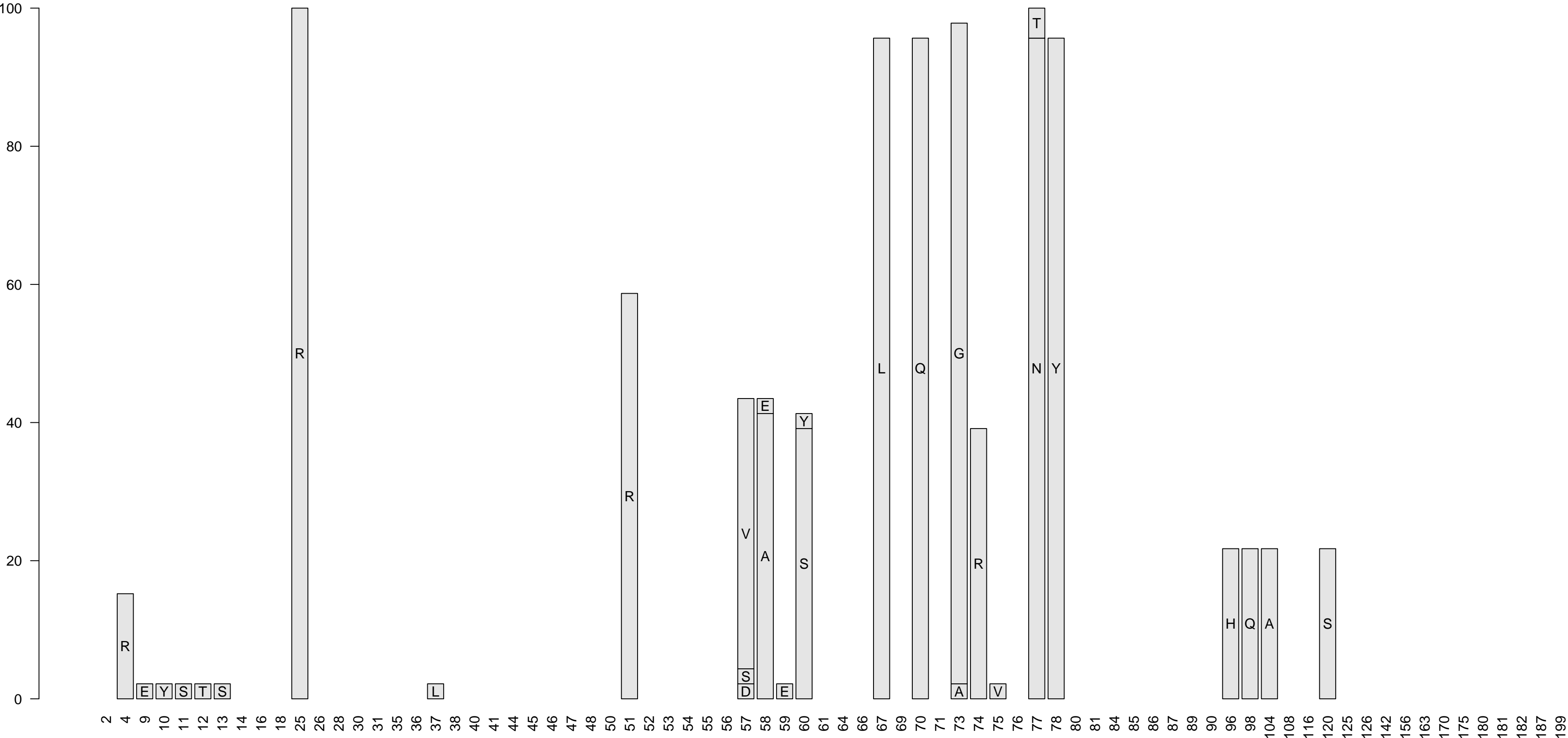

DRB4

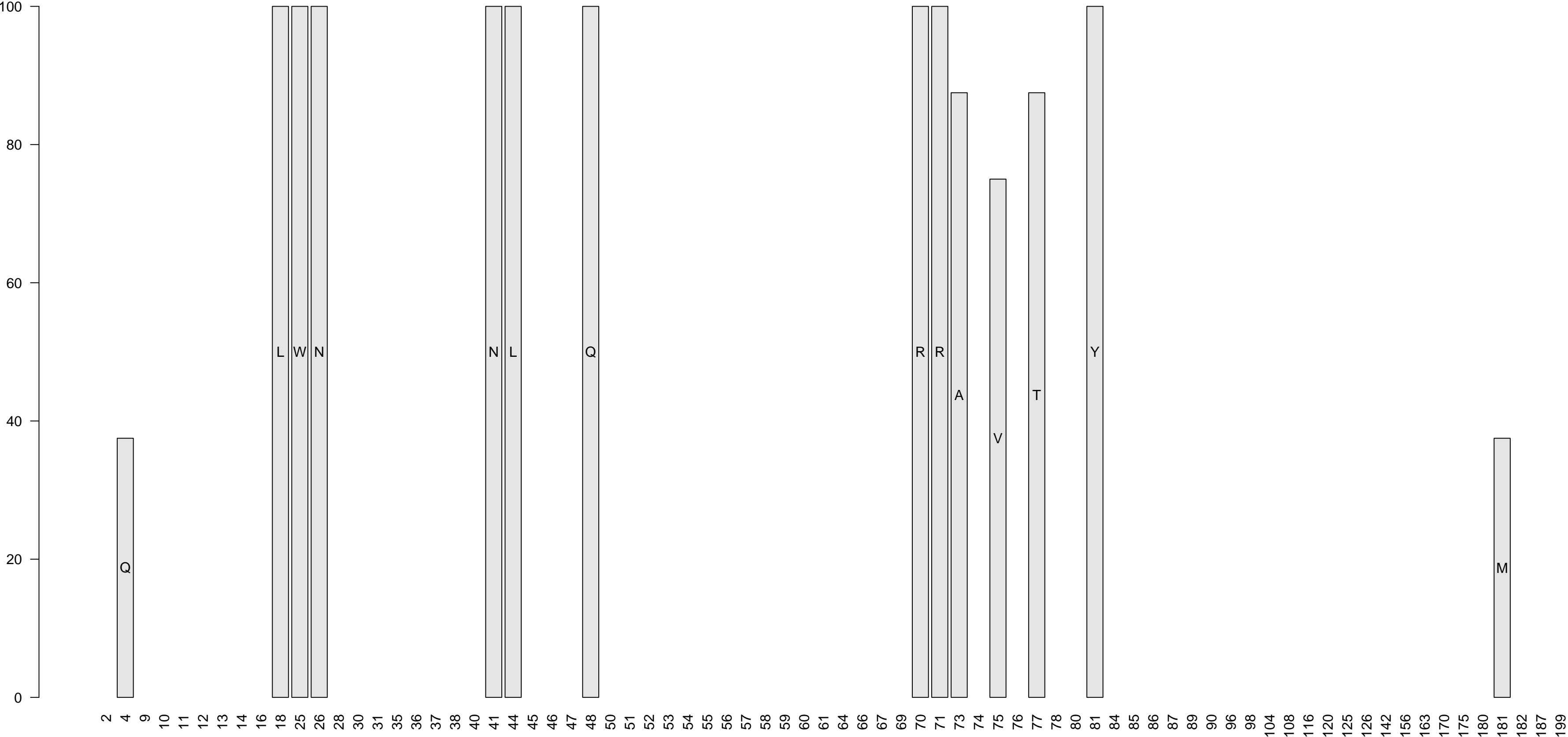

DRB5

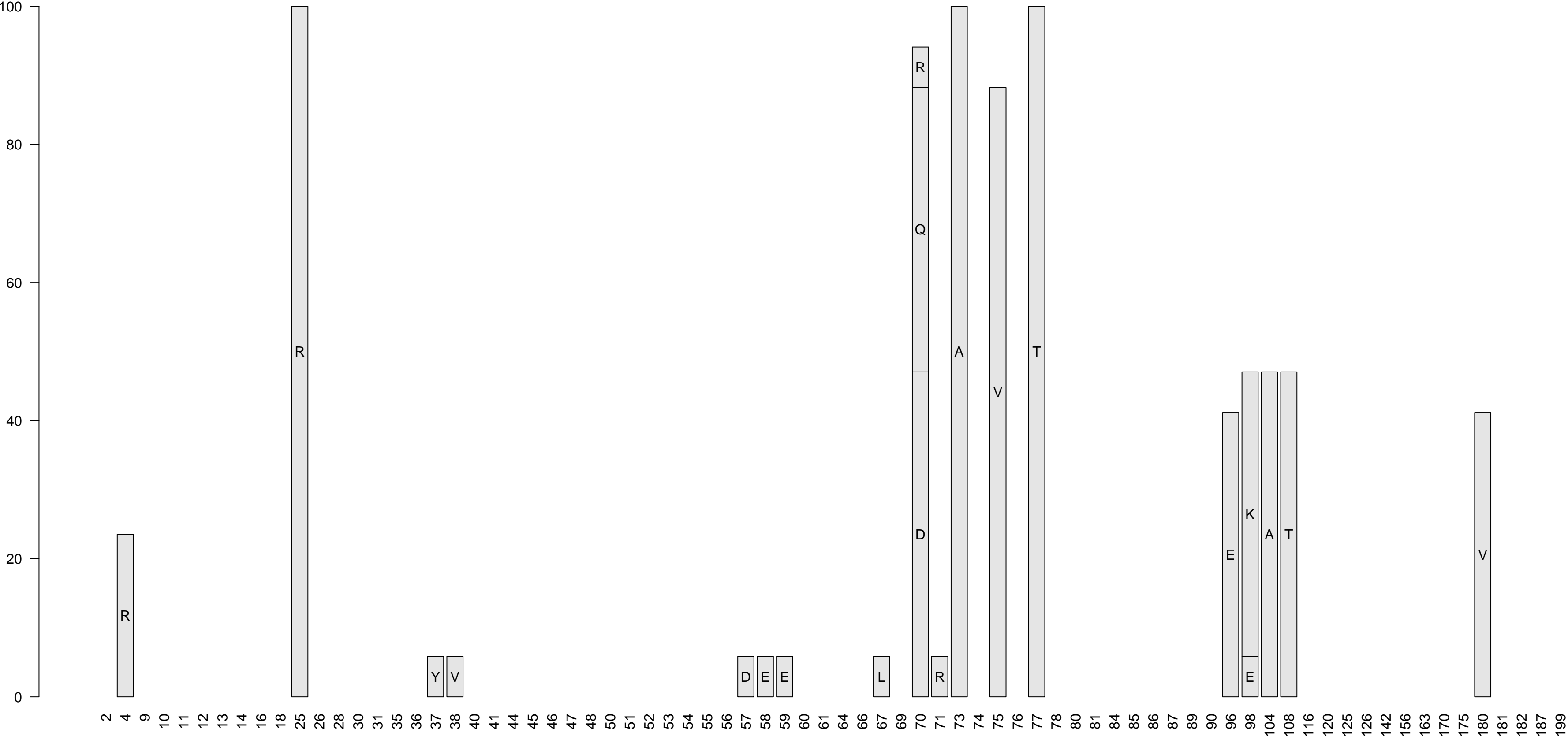

Supplement: Supplementary file 2 [file DataSheet1.PDF]
