# Supplementary material for: epiArt: a graphical HLA eplet amino acid repertoire translation reveals the need for an epitope driven revision of allele group nomenclature
Source: Front Genet. 2024 Oct 16;15:1449301. doi: 10.3389/fgene.2024.1449301 (PMC11521843; doi:10.3389/fgene.2024.1449301)
Supplement: Supplementary file 3 [file DataSheet2.ZIP › Supplementary file 2.html]

HLA-A disparity graphs


# HLA-A disparity graphs

## A\*01

visNetwork


---

## A\*02

visNetwork


---

## A\*03

visNetwork


---

## A\*11

visNetwork


---

## A\*23

visNetwork


---

## A\*24

visNetwork


---

## A\*25

visNetwork


---

## A\*26

visNetwork


---

## A\*29

visNetwork


---

## A\*30

visNetwork


---

## A\*31

visNetwork


---

## A\*32

visNetwork


---

## A\*33

visNetwork


---

## A\*34

visNetwork


---

## A\*36

visNetwork


---

## A\*43

visNetwork


---

## A\*66

visNetwork


---

## A\*68

visNetwork


---

## A\*69

visNetwork


---

## A\*74

visNetwork


---

## A\*80

visNetwork


---
