# Supplementary material for: epiArt: a graphical HLA eplet amino acid repertoire translation reveals the need for an epitope driven revision of allele group nomenclature
Source: Front Genet. 2024 Oct 16;15:1449301. doi: 10.3389/fgene.2024.1449301 (PMC11521843; doi:10.3389/fgene.2024.1449301)
Supplement: Supplementary file 3 [file DataSheet2.ZIP › Supplementary file 3.html]

HLA-B disparity graphs


# HLA-B disparity graphs

## B\*07

visNetwork


---

## B\*08

visNetwork


---

## B\*13

visNetwork


---

## B\*14

visNetwork


---

## B\*15

visNetwork


---

## B\*18

visNetwork


---

## B\*27

visNetwork


---

## B\*35

visNetwork


---

## B\*37

visNetwork


---

## B\*38

visNetwork


---

## B\*39

visNetwork


---

## B\*40

visNetwork


---

## B\*41

visNetwork


---

## B\*42

visNetwork


---

## B\*44

visNetwork


---

## B\*45

visNetwork


---

## B\*46

visNetwork


---

## B\*47

visNetwork


---

## B\*48

visNetwork


---

## B\*49

visNetwork


---

## B\*50

visNetwork


---

## B\*51

visNetwork


---

## B\*52

visNetwork


---

## B\*53

visNetwork


---

## B\*54

visNetwork


---

## B\*55

visNetwork


---

## B\*56

visNetwork


---

## B\*57

visNetwork


---

## B\*58

visNetwork


---

## B\*59

visNetwork


---

## B\*67

visNetwork


---

## B\*73

visNetwork


---

## B\*78

visNetwork


---

## B\*81

visNetwork


---

## B\*82

visNetwork


---

## B\*83

visNetwork


---
