# Supplementary material for: epiArt: a graphical HLA eplet amino acid repertoire translation reveals the need for an epitope driven revision of allele group nomenclature
Source: Front Genet. 2024 Oct 16;15:1449301. doi: 10.3389/fgene.2024.1449301 (PMC11521843; doi:10.3389/fgene.2024.1449301)
Supplement: Supplementary file 3 [file DataSheet2.ZIP › Supplementary file 4.html]

HLA-C disparity graphs


# HLA-C disparity graphs

## C\*01

visNetwork


---

## C\*02

visNetwork


---

## C\*03

visNetwork


---

## C\*04

visNetwork


---

## C\*05

visNetwork


---

## C\*06

visNetwork


---

## C\*07

visNetwork


---

## C\*08

visNetwork


---

## C\*12

visNetwork


---

## C\*14

visNetwork


---

## C\*15

visNetwork


---

## C\*16

visNetwork


---

## C\*17

visNetwork


---

## C\*18

visNetwork


---
