# Supplementary material for: epiArt: a graphical HLA eplet amino acid repertoire translation reveals the need for an epitope driven revision of allele group nomenclature
Source: Front Genet. 2024 Oct 16;15:1449301. doi: 10.3389/fgene.2024.1449301 (PMC11521843; doi:10.3389/fgene.2024.1449301)
Supplement: Supplementary file 3 [file DataSheet2.ZIP › Supplementary file 5.html]

HLA-DRB1 disparity graphs


# HLA-DRB1 disparity graphs

## DRB1\*01

visNetwork


---

## DRB1\*03

visNetwork


---

## DRB1\*04

visNetwork


---

## DRB1\*07

visNetwork


---

## DRB1\*08

visNetwork


---

## DRB1\*09

visNetwork


---

## DRB1\*10

visNetwork


---

## DRB1\*11

visNetwork


---

## DRB1\*12

visNetwork


---

## DRB1\*13

visNetwork


---

## DRB1\*14

visNetwork


---

## DRB1\*15

visNetwork


---

## DRB1\*16

visNetwork


---

## DRB3\*01

visNetwork


---

## DRB3\*02

visNetwork


---

## DRB3\*03

visNetwork


---

## DRB4\*01

visNetwork


---

## DRB5\*01

visNetwork


---

## DRB5\*02

visNetwork


---

## DQA1\*01

visNetwork


---

## DQA1\*02

visNetwork


---

## DQA1\*03

visNetwork


---

## DQA1\*04

visNetwork


---

## DQA1\*05

visNetwork


---

## DQA1\*06

visNetwork


---

## DQB1\*02

visNetwork


---

## DQB1\*03

visNetwork


---

## DQB1\*04

visNetwork


---

## DQB1\*05

visNetwork


---

## DQB1\*06

visNetwork


---
